# Characteristics of a Series of Three Bacteriophages Infecting *Salmonella enterica* Strains

**DOI:** 10.3390/ijms21176152

**Published:** 2020-08-26

**Authors:** Katarzyna Kosznik-Kwaśnicka, Karolina Ciemińska, Michał Grabski, Łukasz Grabowski, Marcin Górniak, Agata Jurczak-Kurek, Grzegorz Węgrzyn, Alicja Węgrzyn

**Affiliations:** 1 Laboratory of Molecular Biology, Institute of Biochemistry and Biophysics, Polish Academy of Sciences, Kładki 24, 80-822 Gdansk, Poland; katarzyna.kwasnicka@biol.ug.edu.pl (K.K.-K.); lukas.grabowski95@gmail.com (Ł.G.); 2 Department of Molecular Biology, University of Gdansk, Wita Stwosza 59, 80-308 Gdansk, Poland; karolina.cieminska@gmail.com (K.C.); michal.grabski@phdstud.ug.edu.pl (M.G.); grzegorz.wegrzyn@biol.ug.edu.pl (G.W.); 3 Institute of Oceanology, Polish Academy of Sciences, Powstańców Warszawy 55, 81-712 Sopot, Poland; 4 Department of Molecular Evolution, University of Gdansk, Wita Stwosza 59, 80-308 Gdansk, Poland; marcin.gorniak@ug.edu.pl (M.G.); agata.jurczak-kurek@ug.edu.pl (A.J.-K.)

**Keywords:** bacteriophages, *Salmonella*, lytic development, genomic analysis

## Abstract

Molecular and functional characterization of a series of three bacteriophages, vB_SenM-1, vB_SenM-2, and vB_SenS-3, infecting various *Salmonella enterica* serovars and strains is presented. All these phages were able to develop lytically while not forming prophages. Moreover, they were able to survive at pH 3. The phages revealed different host ranges within serovars and strains of *S. enterica,* different adsorption rates on host cells, and different lytic growth kinetics at various temperatures (in the range of 25 to 42 °C). They efficiently reduced the number of cells in the bacterial biofilm and decreased the biofilm mass. Whole genome sequences of these phages have been determined and analyzed, including their phylogenetic relationships. In conclusion, we have demonstrated detailed characterization of a series of three bacteriophages, vB_SenM-1, vB_SenM-2, and vB_SenS-3, which reveal favorable features in light of their potential use in phage therapy of humans and animals, as well as for food protection purposes.

## 1. Introduction

The problem of infections caused by antibiotic-resistant bacteria is global [1]. The appearance of bacterial strains resistant to most or even all known antibiotics has become a real difficulty in medicine [2,3], and the need to find alternative methods of treatment of human and animal diseases caused by such bacteria is obvious [4,5]. The use of bacteriophages (or phages), viruses infecting bacterial cells, as alternative therapeutic agents is currently a hot topic; however, apart from many advantages of the use of phages in the treatment of humans and animals, including specificity of phages to selected bacteria without affecting the natural microbiota, their propagation restricted only to the presence of hosts, possibility to kill antibiotic-resistant cells, and a lack of documented considerable adverse effects related to phage administration, there are also controversies about both the efficacy and safety of such a method [6].

When considering phage therapy as an alternative treatment of human and animal infectious diseases, some conditions and requirements appear crucial [6,7,8]. Firstly, a large collection of phages infecting various bacterial species and strains must be established to offer an effective means of treatment of patients or animals suffering from diseases caused by different bacteria. Secondly, lytic rather than temperate bacteriophages should be used to achieve effective killing of bacterial pathogens and to avoid formation of lysogens which become resistant to phages. Thirdly, phages used in therapeutic approaches should not bear genes coding for toxins or other agents which are deleterious for humans and animals. These requirements indicate that isolation and detailed characterization of many bacteriophages is necessary to develop potentially effective phage therapy procedures [6,7,8].

*Salmonella enterica* is a foodborne pathogen, with poultry derived products being the main transmission factor to humans [9]. *S. enterica* can live in the chicken gastrointestinal tract and in most cases it does not cause any illness symptoms. However, depending on the type of serovar and the composition of the chicken’s gut microflora, it can quickly spread, resulting in later contamination in the abattoir during the process of meat preparation [10]. Serotypes most commonly responsible for the disease in humans are *S.* Typhimurium and *S.* Enteritidis. Other serotypes may vary depending on such factors as economy and the geography of a specific region [11]. As detection of *S. enterica* serotypes Typhimurium and Enteritidis results in many countries in immediate liquidation of the flock and legal restrictions are pushing towards prohibiting the use of antibiotics in farming animals, alternative methods of treatment—phages, in this case—are being tested for animal and food protection [9,12].

In fact, many papers have been published recently which described bacteriophages that might be used in phage therapy against *Salmonella*. They include reports on characterization of previously unknown bacteriophages having the potential for use in phage therapy [13,14,15,16,17,18,19,20], experimental studies with bacteriophages applied to poultry [21,22,23,24,25,26], and the use of bacteriophages in experimental phage therapy in mouse [27,28,29,30] or pig [31] models. Although promising results were obtained in these studies, and economic analyses have been performed to assess costs and benefits of the use of phage therapy for the control of *Salmonella* in poultry [9,32], it is evident that the host range of the vast majority of *Salmonella* phages is restricted to specific strains or serovars. Therefore, characterization of more bacteriophages infecting various *Salmonella* serovars and creation of large collections of different bacteriophages capable of killing such differential hosts appears to be necessary for the introduction of effective anti-*Salmonella* phage therapy.

The aim of this work was to characterize three phages infecting strains of *S. enterica*, a pathogen of humans and animals. These phages were isolated previously from urban sewage and preliminarily characterized, including classification, sizes of virions, and plaque morphology and size [33]. It appeared that, although infecting the same bacterial species, they can differ in many aspects of the optimal conditions for lytic development; therefore, each of them might be used for different purposes, as food protection or treatment of humans or animals. Hence, these phages might be potential tools in the development of anti-*Salmonella* therapies, and their detailed characterization is presented in this report.

## 2. Results

### 2.1. Morphology of Phage Virions and Plaque Morphology

Virion dimensions of vB_SenM-1, vB_SenM-2, and vB_SenS-3 were reported previously, as were morphologies of plaques [33]. Here, we present electron micrographs of vB_SenM-1, vB_SenM-2, and vB_SenS-3 virions which were not published earlier (Supplementary Figure S1).

### 2.2. Lysogenization Ability Assessment

To assess whether vB_SenM-1, vB_SenM-2, and vB_SenS-3 are able to form prophages, lysogenization tests were performed as described in Section 4.10. However, among bacteria which survived infection by tested phages, no lysogens could be identified as no plaques were observed after the induction of phage-treated bacteria with mitomycin C. Therefore, these results indicate that vB_SenM-1, vB_SenM-2, and vB_SenS-3 are virulent phages, unable to lysogenize host cells. This was additionally confirmed by analysis of phage genomes, as described in Section 2.8, where no lysogenization-specific genes could be detected in these genomes.

### 2.3. Host Range and Adsorption on Host Cells

To determine the host range of phages vB_SenM-1, vB_SenM-2, and vB_SenS-3, we have employed a collection of *S. enetrica* strains representing various serovars. All these strains are clinical isolates; thus, performed tests might indicate the potential usefulness of investigated bacteriophages in phage therapy. As demonstrated in Table 1, there are various host ranges for each tested bacteriophage. However, most of the investigated *S. enetrica* strains were sensitive to at least one phage, with only one strain, *S.* Virchow, resistant to vB_SenM-1, vB_SenM-2, and vB_SenS-3. Such profiles of host sensitivities make the tested phages promising in light of their potential use in phage therapy.

**Figure 1:**
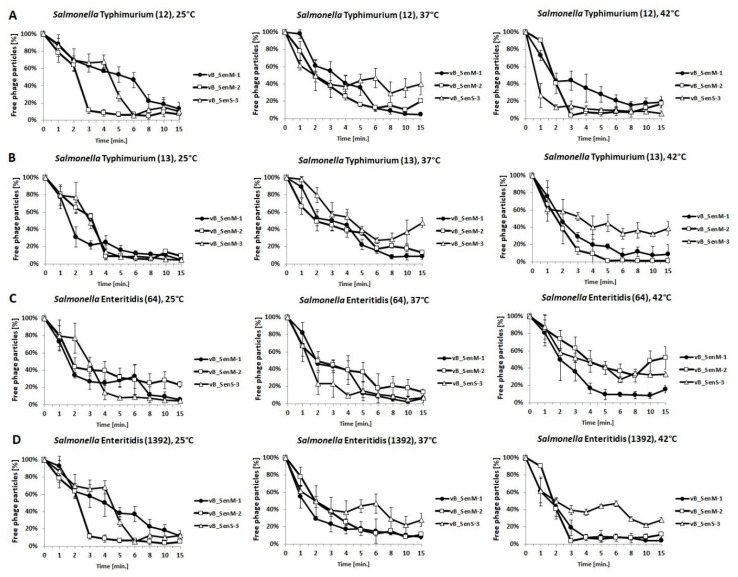
Adsorption rate of phages vB_SenM-1 (closed circles), vB_SenM-2 (open squares), and vB_SenS-3 (open triangles) on *S.* Typhimurium (**A**,**B**) and *S.* Enteritidis (**C**,**D**) at 25, 37, and 42 °C. Number of free phage particles at time 0 was used as reference value (100%). Mean values from three independent experiments are shown, with error bars representing SD.

Efficiency of adsorption of tested phages on host cells was assessed using four selected *S. enterica* strains, two belonging to the Typhimurium serotype and two to Enteritidis serotype. Experiments were performed at three temperatures: 25, 37, and 42 °C. We have shown that titers of all phages were stable after incubation of virions for 60 min at all tested temperatures (Supplementary Table S1), indicating that any observed decrease in the number of free phages in experiments with host strains resulted from the adsorption of virions on bacterial cells rather than from virion inactivation. The most efficient adsorption was found for vB_SenM-1 on *S.* Typhimurium 13 at 25 °C and on *S.* Enteritidis 1392 at 42 °C, vB_SenM-2 on *S.* Typhimurium 12 at 25 °C and on *S.* Enteritidis 1392 at 25 and 42 °C, and vB_SenS-3 on Typhimurium 12 at 43 °C and on *S.* Enteritidis 64 at 37 °C (Figure 1). Nevertheless, all investigated phages adsorbed efficiently on bacterial cells within 15 min (Figure 1). These results indicated that the first stage of infection by vB_SenM-1, vB_SenM-2, and vB_SenS-3 was effective.

### 2.4. One-Step Growth Experiments

**Figure 2:**
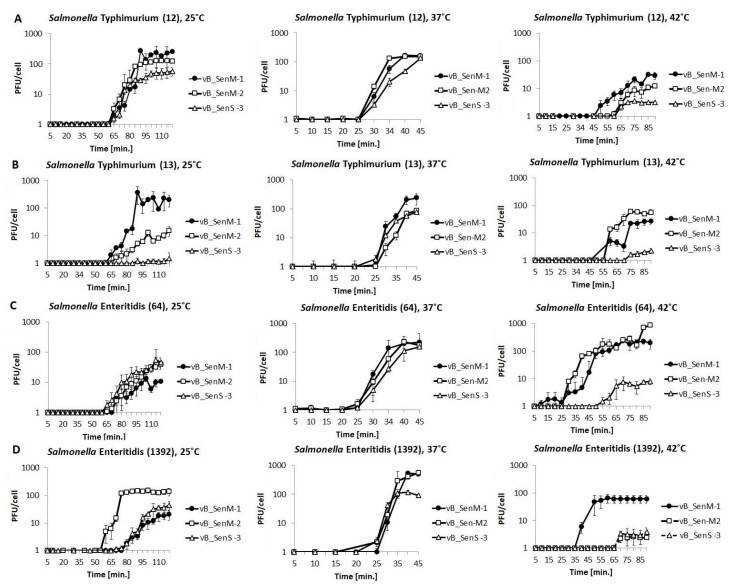
One-step growth experiments with phages vB_SenM-1 (closed circles), vB_SenM-2 (open squares), and vB_SenS-3 (open triangles) on *S.* Typhimurium (**A**,**B**) and *S.* Enteritidis (**C**,**D**) at 25, 37, and 42 °C, at m.o.i = 1. Mean values from three independent experiments are shown, with error bars representing SD.

To assess the kinetics of intracellular phage development, one-step growth experiments were performed. As in phage adsorption experiments, four *S. eneterica* strains were used as hosts, and experiments were performed at 25, 37, and 42 °C. A temperature of 37 °C appeared to be optimal for all tested phages (Figure 2), which allowed the most efficient propagation of phages on all investigated hosts, giving phage yield between 58 and 499 per infected cell, on average (Table 2). The tested phages could also develop at either 25 or 42 °C, but under these conditions, the development was less effective in various host strains. For example, vB_SenS-3 developed poorly at 25 and 42 °C in *S.* Typhimurium 13 and *S.* Enteritidis 64, and lytic growth of both vB_SenM-2 and vB_SenS-3 was impaired in *S.* Enteritidis 1392 (Figure 2, Table 2). Nevertheless, each investigated *S. enterica* strain could be efficiently infected with at least one assessed phage at various temperatures, with the optimal temperature being 37 °C. These results were confirmed in independent experiments where PFU/mL of each phage was determined at 25, 37, and 42 °C and at multiplicity of infection (m.o.i.) 1, 0.5, and 0.1 (Supplementary Figures S2–S4).

### 2.5. Lysis Profile

**Figure 3:**
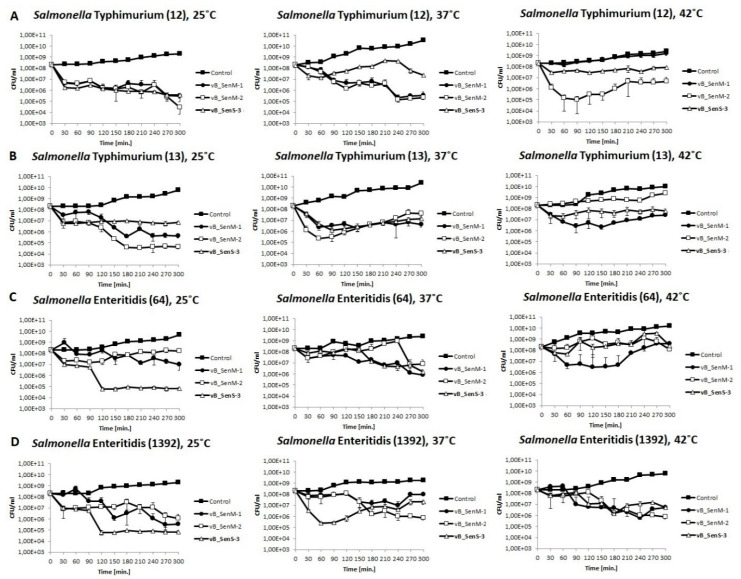
Changes in CFU/mL values of bacterial cultures of *S.* Typhimurium (**A**,**B**) and *S.* Enteritidis (**C**,**D**) infected with phages vB_SenM-1 (closed circles), vB_SenM-2 (open squares), and vB_SenS-3 (closed triangles) at m.o.i = 1, compared with uninfected control (closed squares). Mean values from three independent experiments are shown, with error bars representing SD.

We have tested lysis profiles of the four bacterial strains (two *S.* Typhimurium strains and two *S.* Enteritidis strains) by all investigated phages at 25, 37, and 42 °C and at different m.o.i. Bacterial growth was monitored by determination of CFU/mL. Results of experiments performed at m.o.i. = 1 are presented in Figure 3, and results of those conducted at m.o.i. 0.5 or 0.1 are shown in Supplementary Figures S5 and S6. In addition, measurement of OD_600_ of bacterial cultures was monitored under the same conditions (Supplementary Figures S7–S9). These experiments generally corroborated the conclusions made on the basis of one-step growth experiments and indicated that the number of living bacterial cells of each of the host strains could be efficiently reduced by at least one of the tested phages at every assessed temperature. There were some ostensible discrepancies between results of one-step growth experiments and bacterial culture growth as assessed by measurement of OD_600_ (phages vB_SenM-1 and vB_SenM-2 gave high bursts size on *S.* Enteriditis 1392 at 37 °C and on *S.* Enteriditis 64 at 42 °C, while under these conditions, OD_600_ values resemble those of uninfected cultures). They might arise because, in one-step growth, we used chloroform to liberate phages from cells; thus, OD_600_ of bacterial cultures might in some cases remain relatively high if natural cell lysis is delayed.

### 2.6. pH Sensitivity

Sensitivity of the tested phages to various pH conditions was preliminarily assessed previously [13]. However, here, we investigated the stability of the phages under conditions of extremely low pH in more detail. Therefore, we have tested the effects of incubation of virions of vB_SenM-1, vB_SenM-2, and vB_SenS-3 in Lysogeny Broth (LB) medium with pH between 1 and 3. This can be important for the potential use of studied bacteriophages in phage therapy, as oral administration of phages leads to their exposition to acidic environment in the stomach. Although the use of pH below 2 resulted in effective inactivation of all tested phages, vB-SenM-1, vB_SenM-2, and vB_SenS-3 could survive 1-h incubation at pH 3 at the levels of 3.2%–5.7%, 6.3%–18.3%, and 6.2%–13.3%, respectively, depending on temperature (Table 3).

### 2.7. Effects of Phages on Bacterial Biofilm

**Figure 4:**
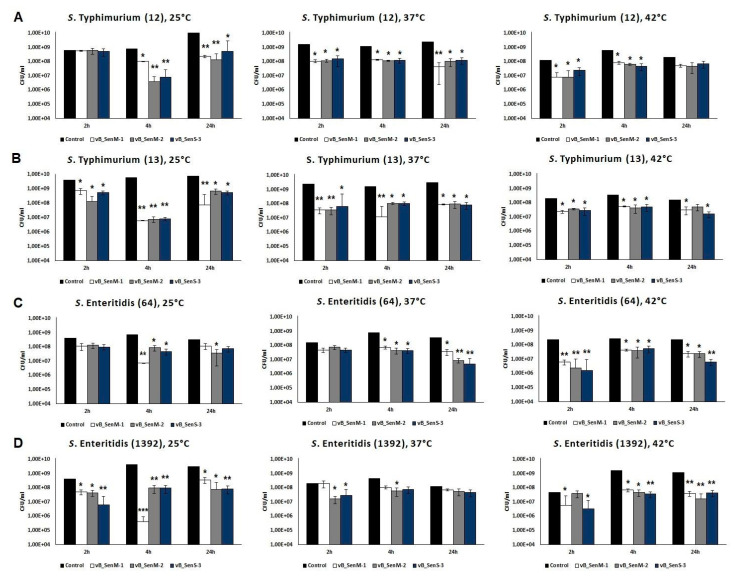
Reduction in the number of bacteria (*S. enterica* serotypes Typhimurium (**A**,**B**) and Enteritidis (**C**,**D**)) from biofilm by phages vB_SenM-1 (white bars), vB_SenM-2 (grey bars), and vB_SenS-3 (navy blue bars) at 25, 37, and 42 °C. CFU/mL of bacterial biofilm untreated with bacteriophages was used as control and reference value (100%). Mean values from three independent experiments are shown, with error bars representing SD. Statistical analysis was performed using *t*-test; *p* < 0.05 (*), *p* < 0.01 (**).

**Figure 5:**
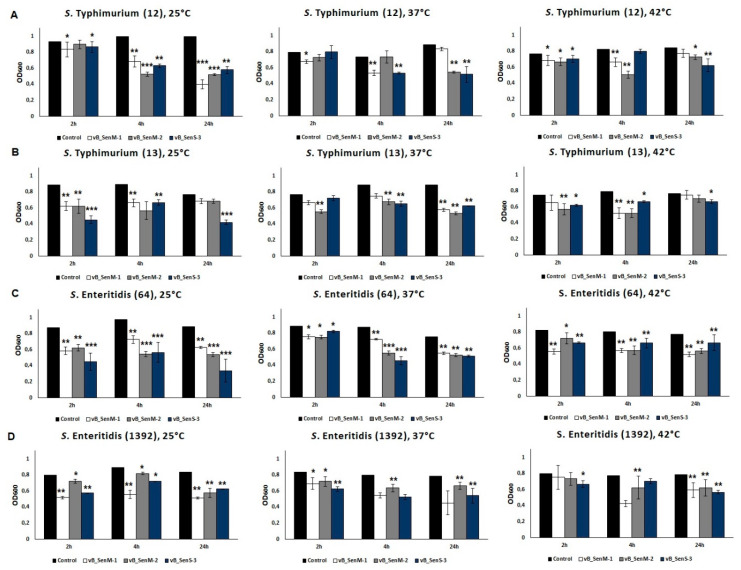
Changes in biofilm mass of different *S. enterica* serotypes Typhimurium 12 (**A**), 13 (**B**) and Enteritidis 64 (**C**), 1392 (**D**) caused by phages vB_SenM-1, vB_SenM-2, and vB_SenS-3 after 4 h of incubation at 25, 37, or 42 °C. Mean values from three independent experiments are shown, with error bars representing SD. Statistical analysis was performed using *t*-test; *p* < 0.05 (*), *p* < 0.01 (**), *p* < 0.001 (***).

The efficiency of reducing the number of bacterial cells included in a biofilm by phages vB_SenM-1, vB_SenM-2, and vB_SenS-3 was tested. We found that each tested phage was able to eliminate 47%–99% of bacterial cells (depending on the host and temperature) from the biofilm formed by any of the four tested *S. enterica* strains (Figure 4). This phage activity occurred at all tested temperatures. The optical density of biofilms after phage treatment is presented in Figure 5. A decrease in the biofilm mass was evident after treatment by phages vB_SenM-1, vB_SenM-2, and vB_SenS-3.

### 2.8. Analysis of Phage Genomes

The whole genomes of phages vB_SenM-1 and vB_SenS-3 have been sequenced. The genome of phage vb_SenM-2 has been described previously [33], and it is deposited in GenBank (accession number: KX171211).

**Figure 6:**
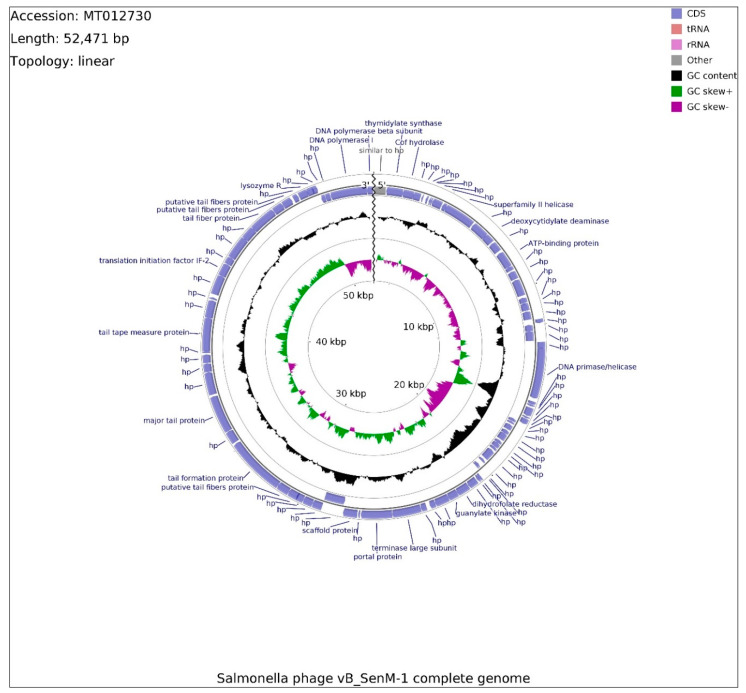
Schematic linear map of phage vB_SenM-1 genome (ends of the genome are indicated by the wavy line). The inner rings show genome location, GC skew + (green) and — (purple) and GC content (black). Two the most external rings show identified open reading frames (blue arrows) and results of genome annotation process.

The genome of phage vB_SenM-1 contains 52,471 bp, with overall GC content of 45.9%, and is presented in the linear topology in Figure 6. Determination of open reading frames (ORFs) distinguished 87 putative protein-coding genes, among which 22 might be classified as known proteins (similar to previously reported ones) and 65 as putative. In total, 47 ORFs are located on the leading strand and 40 on the complementary strand.

Out of 87 ORFs, 76 initiate with the ATG start codon, whereas GTG and TTG occur less frequently, seven and four times, respectively. Stop codons were found to be more variable, from the most frequently observed codon TAA (46), throughout TGA (32), to the least frequent TAG (9). The total number of functionally assigned ORFs (18) was enriched by domain analysis, revealing four hypothetical proteins bearing domains, three of which are putatively engaged in bacteriophage morphogenesis (vB_SenM-1_68, vB_SenM-1_76, vB_SenM-1_79). Furthermore, ORFs were divided into four functional groups due to their assigned functions: morphogenesis (8), lysis (1), DNA packaging (3), and DNA replication (4), assembled in a tile-like manner. The lack of genes unambiguously related to lysogeny suggests that the phage is virulent; however, it was impossible to classify the life cycle of vB_SenM-1 phage based solely on the genome analysis. Besides hypothetical proteins with domains of tail chaperone function (vB_SenM-1_68) and baseplate J-like protein (vB_SenM-1_76), the rest of the morphogenesis ORFs are putatively engaged in tail assembly (vB_SenM-1_62, vB_SenM-1_63, vB_SenM-1_65, vB_SenM-1_78, and vB_SenM-1_79) and with tail tape-measure protein (vBSenM-1_70). No capsid proteins were identified. This section of the genome encompasses OFR74, potentially coding for translation initiation factor IF-2, which also bears a domain putatively involved in the process of “opening” the host cell membrane (vB_SenM-1_74). Coding DNA sequences (CDSs) involved in DNA packaging consist of genes for the terminase large subunit, portal and scaffold proteins located within 23869-27772 bp span. DNA replication encompasses four CDSs, two polymerase subunits (vB_SenM-1_87 and 88) within close proximity, and two CDSs scattered around the genome putatively coding for helicase (vB_SenM-1_11) and primase/helicase (vB_SenM-1_27). This section is intersected by a sequence similar to the gene of hypothetical protein (vBSenM1_01) missing a stop codon, which is typically located downstream of DNA polymerase CDSs (MT074444.1, MK770411.1). Using the PhageTerm software and Li’s method, the COS packaging strategy was identified for this linear genome. The sequences of the genome of phage vB_SenM-1 have been deposited in GenBank (accession number: MT012730.1).

**Figure 7:**
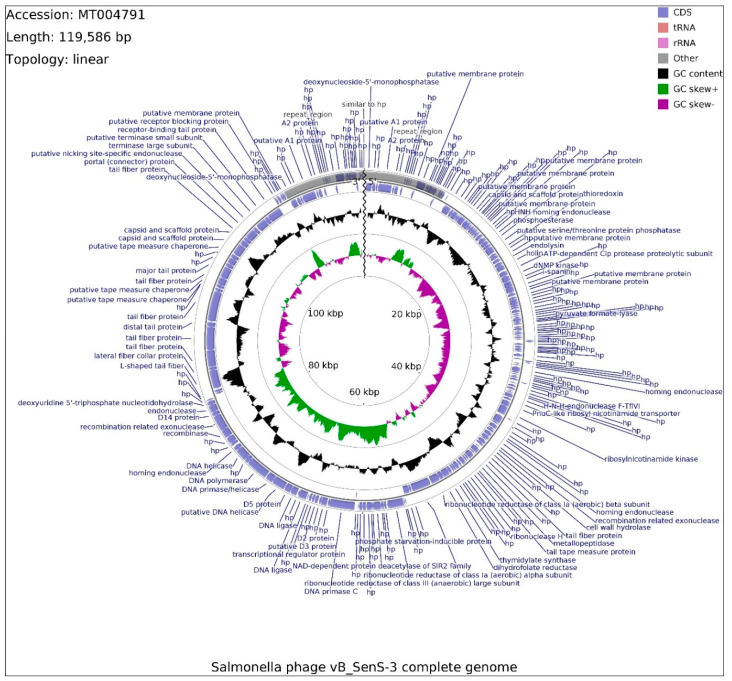
Schematic linear map of genome of the vB_SenS-3 phage (ends of the genome are indicated by the wavy line). The inner rings show genome location, GC skew + (green) and—(purple) and GC content (black). The two most external rings show identified open reading frames (blue arrows) and results of genome annotation process. Repeated sequence at the beginning and the end of phage genome is marked in gray.

The genome of phage vB_SenS-3 consists of 119,586 bp, with overall GC content of 39.97% and a linear topology with repeat regions (9455 bp) located at sequence ends. The genome was found to be circularly permutated with redundant ends. The linear map of this genome is presented in Figure 7.

Determination of ORFs distinguished 229 putative protein-coding genes, of which 23 are embedded in the repeat region. Within the total ORFs, 78 are located on the leading strand and 151 are located on the complementary strand. Functions of 83 proteins could be determined on the basis of similarity to previously published sequences, and 146 remain putative. Out of 229 ORFs, 185 initiate with the ATG start codon, whereas GTG and TTG occur less frequently, 18 and 26 times, respectively. Frequency of observed stop codons is set out as follows: TAA (158), TGA (46), TAG (25). ORFs with assigned functions mainly use ATG start codons, with the exception of genes coding for holin (vB_SenS3_51) and DNA polymerase (vB_SenS3_167), utilizing GTG and TTG codons, respectively. ORFs with assigned functions were divided into four functional groups: morphogenesis (16), lysis (2), DNA packaging (9), and DNA replication (9). Two CDSs putatively coding for endolysin and holin (vB_SenS3_50 and 51) represent genes involved in host cell lysis. Morphogenesis CDSs are scattered around the genome, mainly gathered within positions 86,460-103,386 bp (8), where three CDSs code for head, capsid, and scaffold proteins (vB_SenS3_194, vB_SenS3_195, vB_SenS3_196). CDS with similar function was found in position vB_SenS3_32, with a significantly shorter sequence. The rest of the morphogenesis-related CDSs are involved in tail assembly, while the vB_SenS3_186 sequence was found to encompass Rho-independent transcription terminator. The DNA packaging group contains endonucleases (vB_SenS3_116, vB_SenS3_170, vB_SenS3_176), ribonuclease H (vB_SenS3_129), with portal protein (vBSenS3_198) and terminase large and small subunits (vB_SenS3_200, vB_SenS3_201), located upstream of the morphogenesis-related CDS conglomerate. Within positions 66,561-76,182 bp, genes encoding two ligases (vB_SenS3_161, vB_SenS3_163), primase (vB_SenS3_166), DNA polymerase (vB_SenS3_167), and DNA helicase (vB_SenS3_165, vB_SenS3__69) were found, and all these genes which are putatively responsible for phage DNA replication were predicted to be under the control of the same promoter, 66,350-66,395 bp, with the exception of vB_SenS3_169. The primase gene was found to possess Rho-independent transcription terminator. Additionally, DNA primase C gene (vB_SenS3_153) was found downstream of genes engaged in DNA replication. The vB_SenS3_173 sequence classified as putative recombinase gene was subjected to domain analysis at the amino acid level of the gene product, and the sequence was found to be partially similar to DNA repair exonuclease SbcCD nuclease subunit, responsible for cleaving DNA hairpin structures. The genome of phage vB_SenS-3 has been deposited in GenBank (accession number: MT004791.1).

**Figure 8:**
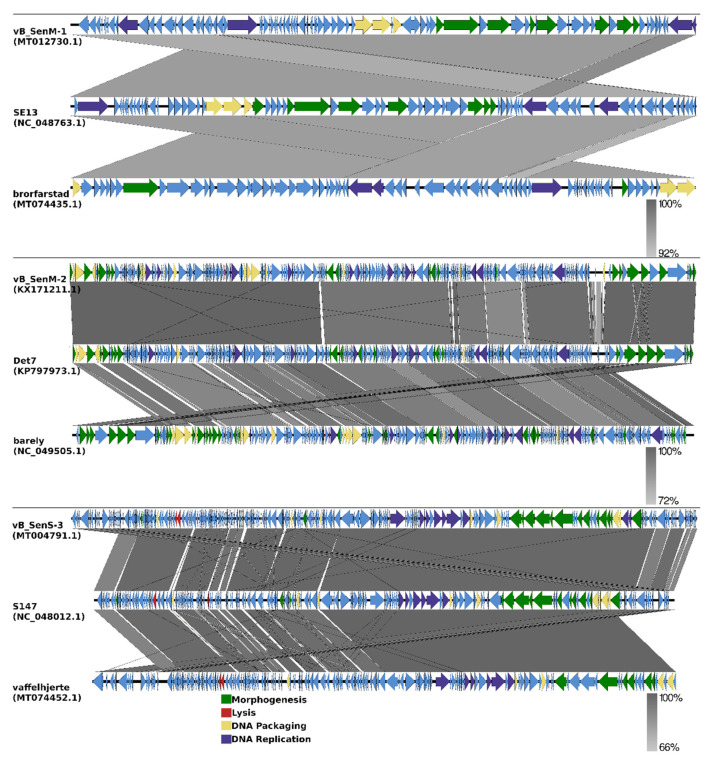
EasyFig output image of the genomic comparison between phages vB_SenM-1, vB_SenM-2, and vB_SenS-3 and the most closely related phages. Phage genomes are presented by linear visualization, with coding regions shown as arrows. Selected open reading frames are colored in relation to their functions. The percentage of sequence similarity is indicated by the intensity of the gray color.

Annotations of genomes of phages vB_SenM-1 and vB_SenS-3 are presented in Supplementary Tables S2 and S3, respectively. In addition, genomic comparisons between phages vB_SenM-1, vB_SenM-2, and vB_SenS-3 and the most closely related phages are shown in Figure 8 (see also Section 2.9).

### 2.9. Phylogenetic Analyses of the Phages

**Figure 9:**
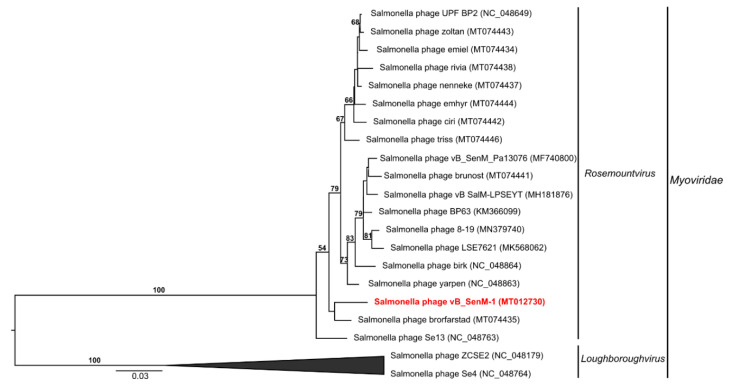
Neighbor-joining phylogenetic tree of the terminase large subunit (TerL) gene nucleotide sequences data showing the phylogenetic position of phage vB_SenM-1 (in red color) within *Rosemountvirus*. The reference sequences were collected from NCBI database. The tree was constructed using PAUP* with GTR + G+ I model of DNA substitution. Bootstrap values >50%, calculated based on 1000 resamplings, are shown above branches.

**Figure 10:**
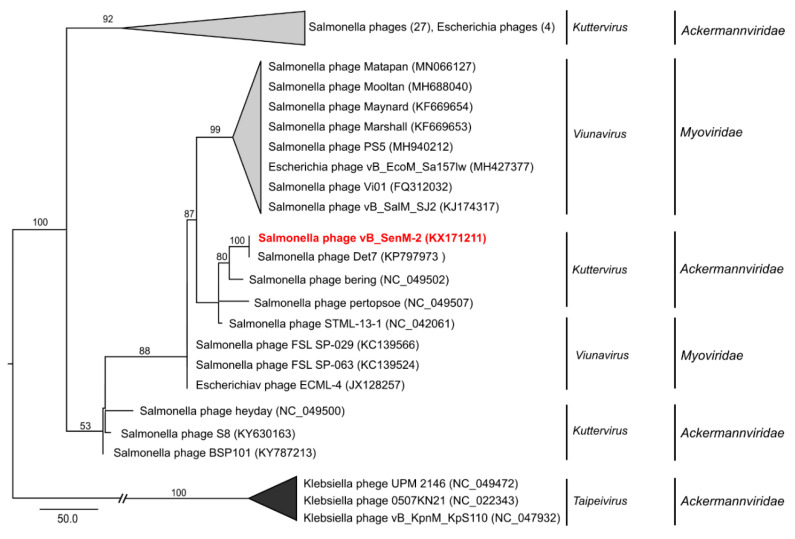
Neighbor-joining phylogenetic tree of the terminase large subunit (TerL) gene nucleotide sequences data showing the phylogenetic position of phage vB_SenM-2 (in red color) within *Kuttervirus*. The reference sequences were collected from NCBI database. The tree was constructed using PAUP* with GTR + G+ I model of DNA substitution. Bootstrap values >50%, calculated based on 1000 resamplings, are shown above branches.

**Figure 11:**
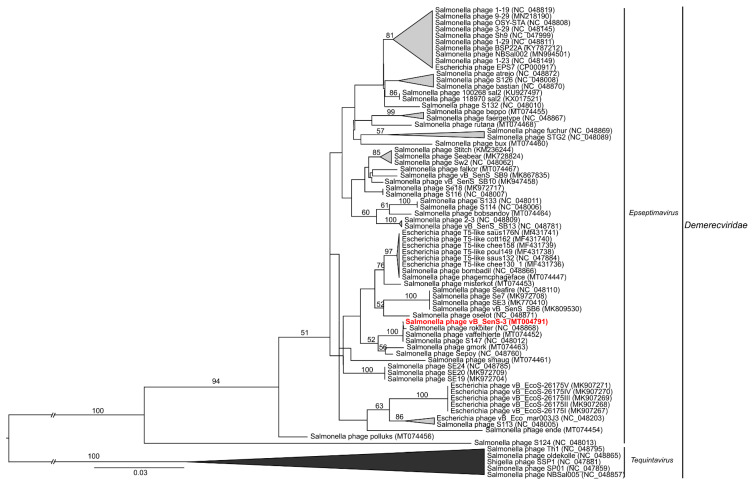
Neighbor-joining phylogenetic tree of the terminase large subunit (TerL) gene nucleotide sequences data showing the phylogenetic position of phage vB_SenS-3 (in red color) within Epseptimavirus. The reference sequences were collected from NCBI database. The tree was constructed using PAUP* with GTR + G+ I model of DNA substitution. Bootstrap values >50%, calculated based on 1000 resamplings, are shown above branches.

To analyze phylogenetic relationships between investigated phages and other viruses, we have compared the nucleotide sequences of genes coding for large terminase subunits of phages vB_SenM-1, vB_SenM-2, and vB_SenS-3 with the sequences of corresponding genes of other phages (Figure 9, Figure 10, and Figure 11, respectively).

As shown in Figure 9, the sequence of the gene encoding the large terminase subunit of vB_SenM-1 bacteriophage presents the highest identity with that of *Salmonella* phage brorfarstad (MT074435). Sequence similarity searches revealed that the aforementioned phages show a very high level of genome sequence identity (~96%). vB_SenM-1 also shows a high level of identity with other *Salmonella* phages such as SE13 (~96%) (NC_048763), yarpen (~95% identity) (NC_048863), brik (~95% identity) (NC_048864), and LSE7621 (~95% identity) (MK568062) (see also Figure 8). All of the phages are classified as belonging to the family *Myoviridae,* genus *Rosemountvirus* [34,35].

The sequence of the gene encoding the large terminase subunit of the phage vB_SenM-2 shows the highest identity with *Salmonella* phage Det7 (KP797973) [36], belonging to the family *Ackermannviridae*, of the genus Kuttervirus, according to the newest update of ICTV (International Committee on Taxonomy of Viruses) [37]. The phages also show 100% identity at the terminase gene level (Figure 10). This is an update of previous data according to which vB_SenM-2 had presented the highest identity with ViO1 (FQ312032) belonging to *Myoviridae* [33,38]. Thus, in this report, we classify the phage vB_SenM-2 as belonging to *Ackermannviridae*.

The nucleotide sequence of the gene encoding the large terminase subunit of vB_SenS-3 shows 100% identity with the sequences of two other phages, *Salmonella* phage vaffelhjerte (MT074452) and *Salmonella* phage S147 (NC_048012), both belonging to the family *Demerecviridae*, of the genus Epseptimavirus [39,40,41] (Figure 11; see also Figure 8). Sequence similarity searches revealed that the aforementioned phages also show a very high level of genome sequence identity (~99%). vB_SenS-3 shows high identity (~99%) with other phages such as S113 (MH370366) and rokbiter (NC_048868) [41].

## 3. Discussion

Phage therapy is one of the most promising alternatives to treat patients suffering from infectious diseases caused by antibiotic-resistant bacteria [42]. However, apart from legal difficulties which currently impair formal possibilities of the common use of phage therapy in clinical practice [43], there are several microbiological issues which must be considered when applying such therapeutic procedures [44]. Particularly, the crucial requirements are as follows: (**i**) the presence of a large collection of bacteriophages, (**ii**) the use of virulent, rather than temperate, phages, (**iii**) the use of phages devoid of genes coding for toxins and other potential deleterious agents in their genomes. Moreover, it is advantageous to use phages which adsorb efficiently on host cells, propagate rapidly, and give relatively high burst size. In addition, phages whose virions are stable under various environmental conditions are valuable.

*S. enterica* is a pathogenic bacterium, often revealing antibiotic resistance, infecting human and animal gastrointestinal tracts; thus, development of phage therapy against this bacterium is desirable. Phages infecting *Salmonella* strains can also be used in controlling this bacterium in poultry [9], as well as in food protection and dishwashing procedures [2,12,45]. However, each of the abovementioned applications require different properties of phages, like various optimal growth temperatures or resistance to different pH conditions.

In this report, we present characterization of a series of three bacteriophages infecting *S. enterica*. These phages have been isolated from urban sewage, but they were described only preliminarily, in light of biodiversity rather than phage therapy [33]. Nevertheless, we found that they have various properties which make them potentially useful agents in therapeutic and food protection purposes. These phages, named vB_SenM-1, vB_SenM-2, and vB_SenS-3, have virion morphologies characteristic for the *Myoviridae* (vB_SenM-1, vB_SenM-2) and *Siphoviridae* (vB_SenS-3) families (Supplementary Figure S1). Importantly, they are virulent phages, as indicated experimentally by their inability to lysogenize host cells. This is corroborated by genomic analyses which demonstrated a lack of genes potentially involved in lysogenization as well as toxin genes or virulence factors that could pose risk to other prokaryotic or eukaryotic cells (Figure 6 and Figure 7; Supplementary Tables S2 and S3). These features make them appropriate candidates for phage therapy and food protection purposes. The vB_SenM-1, vB_SenM-2, and vB_SenS-3 phages revealed different but relatively broad host ranges within the species *S. enterica*, being able to efficiently infect many different serovars and strains (Table 1), which again is an advantage in light of their practical use.

Developmental parameters of vB_SenM-1, vB_SenM-2, and vB_SenS-3 were characterized. All tested phages adsorbed effectively on various host strains within 15 min at different temperatures (Figure 1), demonstrating desirable features for phage therapy and food protection. One of the crucial properties of phages in light of these applications is the ability to perform effective lytic development at various temperatures and at different m.o.i. values. We found flexibility of the tested phages for these features, as demonstrated by one-step growth experiments and lysis profile determination (Figure 2 and Figure 3; Supplementary Figures S2–S9). Although some preferences of particular phages were evident, the crucial message is that effective reduction in the number of cells of different *S. enterica* strains was possible under every tested condition by at least one of the investigated bacteriophages. One should note that our one-step growth experiments did not estimate the precise latent period and classical burst size, defined as the number of phages liberated by spontaneous lysis of an infected bacterium. Regarding the latent period, we have conducted phage adsorption in an ice-bath and then centrifugation at 4 °C; however, we cannot exclude that phage development might proceed to some extent in cells during these 15 min (5 min phage adsorption, followed by 10 min centrifugation). Classical inhibition of phage development at the initial stages of one-step growth experiment by the use of a metabolic toxin like NaN_3_ could not be achieved as we found that *S. enterica* strains did not restart growth after washing such a compound out. Regarding burst size, we have used chloroform to lyse the cells and to estimate number of mature virions at each experimental time point. This allowed us to determine actual yield of phage progeny (both intra and extracellular) but did not fulfill the classical definition of the burst size as the lysis of hosts cells was not spontaneous.

In light of the potential use of the investigated bacteriophages in phage therapy, some level of resistance to pH levels of 3 (Table 3) and higher [33] may also be important, indicating that the use of compound(s) neutralizing acid conditions in human and animal stomach should be sufficient for the successful delivery of phages into the intestine. Moreover, effective reduction of number of cells of *S. enterica* from a biofilm, revealed by all tested phages at all tested temperatures (Figure 4), as well as reduction in the biofilm mass (Figure 5), are additional advantages when considering applications of vB_SenM-1, vB_SenM-2, and vB_SenS-3 in medicine, animal care, and food protection.

Phylogenetic analysis and sequence similarity searches revealed the phage vB_SenM-1 genome to be similar to previously described phage SE13. This phage has been characterized as being of broad host range among various *S. enterica* serotypes as well as having been effectively used as part of an experimental phage cocktail against *Salmonella* infection in food, especially cantaloupe and lettuce [34,46]. Moreover, phage LSE7621, to which phage vB_SenM-1 shows ~95% identity, has also been effectively tested in *S.* Enteritidis biocontrol on lettuce. However, this phage shows narrower host range than phage vB_SenM-1 [35]. Based on available data and experiments performed in this work, it can be assumed that phage vB_SenM-1 has the potential to be effectively used in food protection.

Phylogenetic and genomic analysis allowed the revision of classification based on the morphology of investigated phages. As a result, SenM-2 is now classified as *Ackermannviridae* instead of *Myoviridae* and SenS-3 is classified as *Demerecviridae* instead of *Siphoviridae*. The classification of SenM-1 remains unchanged—*Myoviridae*. In fact, viral classification is very dynamic due to the growing amount of viral genome data being deposited; therefore, it requires regular updates.

In conclusion, we have demonstrated detailed characterization of a series of three bacteriophages, vB_SenM-1, vB_SenM-2, and vB_SenS-3, which reveal favorable features in light of their potential use in phage therapy of humans and animals, as well as for food protection purposes. They are lytic phages, able to adsorb and propagate on various *S. enterica* strains under different conditions of temperature and m.o.i. Virions of these phages can partially (in the range of 3.2%–13.3%) survive 1-h incubation at pH 3.0. These phages can efficiently decrease both the number of *S. enterica* cells in the biofilm and the biofilm mass. Genomic and bioinformatic analyses allowed us to determine the principles of the organizations of the genomes of the investigated phages and to identify the potential functions of ORFs, revealing similarity to previously described and characterized genes coding for proteins involved in various processes during the development of different bacteriophages. Finally, the construction of phylogenetic trees based on DNA sequences led us to classify SenM-1, SenM-2, and SenS-3 as *Myoviridae*, *Ackermannviridae* and *Demerecviridae*, respectively.

## 4. Materials and Methods

### 4.1. Bacterial Strains and Culture Conditions

*Salmonella enterica* strains used in this study come from the National *Salmonella* Centre at Medical University of Gdansk (Gdansk, Poland) and Bacterial Strain and Plasmid Collection at the University of Gdansk. More detailed description of strains used in this study is presented in Table 1.

### 4.2. Bacterial Culture Conditions

Bacteria were cultivated in LB medium at 25, 37, or 42 °C in a water bath at 150 rpm. LB with 1% agar was used for solid media and 0.7% agar was used in top agar for double-layer agar plates. Overnight incubation was performed at 37 °C.

### 4.3. Preparation of Phage Lysates

Phage lysates were prepared as follows: overnight culture of *S.* Heidelberg was diluted in fresh LB medium at a ratio of 1:100 and incubated with shaking at 37 °C until reaching OD_600_ = 0.15. Phages were then added to final m.o.i = 0.25. The culture was further incubated until lysis of bacterial cells was observed (OD_600_ = 0.05). Lysates were centrifugated at 5 min, 3500 g, 4 °C. The pellet was discarded. Lysates were stored at 4 °C with addition of 1% chloroform.

### 4.4. Electron Microscopy

Purification of bacteriophage vB_SenM-1, vB_SenM-2, and vB_SenS-3 particles was carried out by using the cesium chloride density gradient centrifugation method, according to the procedure described previously [47]. Transmission electron microscopy analysis of the phages was performed in the Laboratory of Electron Microscopy, Faculty of Biology, University of Gdansk, Gdansk, Poland. Virions were negatively stained with uranyl acetate; then, micrographs were taken under a Philips CM 100 electron microscope.

### 4.5. Phage Host Range

Host range of bacteriophages was determined using spot-test method described previously, with some modifications [33,48]. Phages were serially diluted in TM buffer (10 mM Tris-HCl pH 7.2., 10 mM MgSO_4_). The dilutions were then overlaid on top of double-layer agar plates with top agar containing 200 µL of bacterial culture, 10 mM CaCl_2_, and 1% casamino acids. Plates were incubated overnight at 37 °C and then scanned for the presence of single phage plaques. Phage titer was then counted, and results are presented relative to values obtained with the *S.* Heidelberg strain.

### 4.6. Influence of Low pH on Phage Viability

Susceptibility of phages to low pH levels was performed as described previously [33], with some modifications. In order to determine how low pH affects the viability of bacteriophages, 100 µL of phage lysate was added to 900 µL of LB at pH: 1.8, 2.0, 2.2, 2.5, 2.8, and 3.0. The samples were mixed and incubated for 1 h at 25, 37, or 42 °C. Afterwards, samples were serially diluted and overlaid on top of LB-agar plates with top agar containing 200 µL of bacterial culture, 10 mM CaCl_2_, and 1% casamino acids. Plates were incubated overnight at 37 °C and then scanned for plaques.

### 4.7. Phage Adsorption to Bacterial Host Cells

The adsorption assay was performed according to the protocol described before [48,49], with some modifications. Adsorption rate of bacteriophages vB_SenM-1, vB_SenM-2, and vB_SenS-3 was analyzed at three different temperatures (25, 37, and 42 °C) and on four *S. enterica* strains: *S.* Typhimurium 12, *S.* Typhimurium 13, *S.* Enteritidis 64, and *S.* Enteritidis 1392 (Table 1). Next, 10^9^ cells were infected with a phage lysate to final m.o.i. = 0.1 and then incubated at given temperatures. At indicated time points, three individual samples per phage were collected and centrifuged at 6000 *g* for 30 s, to sediment the bacterial cells. The supernatant was diluted in TM buffer and assayed for free, unadsorbed phage particles. The number of viruses mixed with bacterial host cells at time 0 was considered 100% non-adsorbed phages. Other values were compared to this sample.

### 4.8. One-Step Growth Experiment

One-step growth experiments were performed as described previously, with some modifications [50]. In brief, *S. enterica* strains *S.* Typhimurium 12, *S.* Typhimurium 13, *S.* Enteritidis 64, and *S.* Enteritidis 1392 were grown at given temperatures (25, 37, or 42 °C) until reaching OD_600_ = 0.2. Then, 10 mL of bacterial culture was centrifuged (4000× *g*, 10 min, 4 °C), and the pellet was suspended in 1 mL of LB medium. Phage particles were added to the host culture at an m.o.i. = 0.5 and allowed to adsorb for 5 min in an ice-bath. The mixture was centrifuged (4000× *g*, 10 min, 4 °C) to remove unadsorbed viruses. Then, 50 µL of the bacterial mixture was added to 25 mL of LB medium (time 0) and cultivated at 25, 37, or 42 °C. The number of infective centers was estimated from samples taken 1, 2.5, and 5 min after infection by plating under permissive conditions. Then, 100 µL sample was collected at given temperatures, mixed with equal volume of chloroform, cleared by centrifugation (6000× *g*, 30 s) and titrated to determine the number of PFU per ml. The plates were incubated at 37 °C overnight. Yield of progeny phages was calculated as the ratio of phage titer to the number of infection centers. This value should not be interpreted as burst size, since, according to the classical definition, burst size indicates the number of phages liberated by spontaneous lysis of an infected bacterium.

### 4.9. Lysis Profile of Host Bacteria after Phage Infection

The experiments were performed in accordance with previously described procedures using four strains of *S.* enterica [51]. In brief, *S.* Typhimurium 12, *S.* Typhimurium 13, *S.* Enteritidis 64, and *S.* Enteritidis 1392 were cultivated to OD_600_ = 0.2 at 25, 37, or 42 °C. Then, phage stock solution was added to the host at an m.o.i. = 0.1, 0.5, or 1. In negative control experiments, bacteria were inoculated with LB medium instead of phage lysate. Bacterial growth was monitored by measuring the OD_600_ in 15 min intervals over period of 300 min. During this experiment, the number of bacterial cells per ml (CFU/mL) and phage titer (PFU/mL) were also determined. To calculate the number of surviving cells after virus infection, 100 µL of bacterial culture was collected at every 30 min, and serial dilutions were made in 0.89% sodium chloride and overlaid onto LB agar plates. The CFU/mL was calculated based on the counted colonies after overnight incubation at 37 °C. To estimate the PFU/mL, samples were taken every 30 min and diluted in TM buffer. The samples were overlaid onto a double-layer agar plate. The phage titer was determined by counting single plaques.

### 4.10. Efficiency of Lysogenization

The experiments were performed in accordance with previously described protocols [52,53], with some modifications. In brief, *S.* Typhimurium 12, *S.* Typhimurium 13, *S.* Enteritidis 64, and *S.* Enteritidis 1392 were cultivated to OD_600_ = 0.2 at 37 °C. Then, 1 mL of bacterial culture was centrifugated (2000× *g*, 10 min, room temperature) and the pellet was resuspended in 1 mL of LB medium containing 10 mM CaCl_2_. After incubation for 5 min at 30 °C, phage lysate was added to m.o.i = 1. In control experiments, LB was added to the culture instead of phage lysate. Bacteria were incubated for 2 h at 30 °C. Serial dilutions were prepared in TM buffer and 40 µL of each dilution was poured onto LB plates. After overnight incubation at 37 °C, 96 colonies were passaged separately each in a well of a 96-well plate with 200 µL of LB medium (if less colonies were visible on the plates all of them were passaged). The plates were incubated with shaking at 37 °C until bacteria reached OD_600_ = 0.2.

In order to test the resistance to superinfection, 50 µL of bacterial culture was mixed with a solution composed of 10 mM CaCl_2_ and 10% casamino acids and spread onto LB agar. Then, 2.5 µL of phage lysate was spotted on top of it. The plates were then incubated overnight at 37 °C and then scanned for plaques. If plaques were not visible, the clone was recognized as resistant to phage. The resistance to infection was determined as a percent of bacteria uninfected by the phage. Each experiment was performed in triplicate.

For estimation of efficiency of lysogenization, 1 µg/mL mitomycin C was added to 150 µL of remaining bacterial culture. The plates were then incubated for another 3 h. Afterwards, 10 µL of chloroform was added, the plates were centrifugated (2000× *g*, 10 min, 4 °C) and 5 µL of top layer was spotted onto double-layer LB agar plates. The plates were incubated overnight at 37 °C. The presence of lysogen was determined by formation of plaque on double-layer agar plate. The efficiency of lysogenization was determined as percent of lysogens among all tested 96 bacterial colonies. The experiment was performed in triplicate.

### 4.11. Effects of Phages on Bacterial Cells in Biofilm

Biofilm cell biomass after phage infection was determined according to the protocols described previously [48,54], with some modifications. The experiment was performed at three different temperatures (25, 37, and 42 °C) and on four *S. enterica* strains: *S.* Typhimurium 12, *S.* Typhimurium 13, *S.* Enteritidis 64, and *S.* Enteritidis 1392. For biofilm cell culture, *S. enterica* was grown at 37 °C or 42 °C in 12-well polystyrene plate with 0.5 × LB medium for 24 h or at 25 °C for 48 h. After the incubation period, the liquid medium containing planktonic cells was removed. The biofilm was washed with 1 mL of distilled water. In the next step, phage lysate was added to each well, except for controls, to the final titer of 10^9^ PFU/well, and then plates were incubated at 25, 37, or 42 °C for 2, 4, and 24 h. In the case of control wells, the medium was added instead of phage lysate. Following incubation, phage lysate was removed, and biofilms were washed with 1 mL of distilled water and then resuspended in 1 mL of distilled water. Serial dilutions were made and overlaid on top of LB-agar medium and left to dry down. The plates were incubated at 37 °C overnight and then scanned for bacterial colonies.

### 4.12. Assessment of Biofilm Biomass by Crystal Violet Staining after Phage Infection

Biofilms were prepared according to the procedure described above. After incubation with phage lysate (added to final titer 10^9^ PFU/well), the liquid medium was removed and surface-attached cells were treated with 1 mL of 0.1% crystal violet solution (Sigma-Aldrich). Plates were incubated in the dark for 20 min at 42 °C. Crystal violet was then carefully removed and biofilms were washed 2 times with 1 mL of distilled H_2_O. Then, biofilms were fixed by additional incubation at 42 °C for 20 min. Afterwards, crystal violet was dissolved by the addition of 0.5 mL of 96% ethanol. The absorbance was measured in a plate reader at 600 nm (VICTOR, PerkinElmer, Waltham, MA, USA) in order to assess biofilm biomass. Biofilms were also photographed following the fixation in order to visualize the differences in biofilm biomass after phage treatment.

### 4.13. Isolation and Sequencing of Phages vB_SenM-1 and vB_SenS-3 Genomes

The DNA isolation was performed as described previously [33]. Briefly, phage lysates were purified using cesium chloride density gradient. Then, 300 µL of purified lysate was treated with DNase and RNase for 30 min at 37 °C to digest the exogenous bacterial RNA and DNA. After thermal inactivation, the suspension was treated with proteinase K for 60 min at 37 °C. The phage genome DNA was isolated by using the MasterPure™ Complete DNA and RNA Purification Kit (Epicentre) in accordance with the manufacturer’s protocol. DNA concentration was determined spectrophotometrically by measuring the absorbance at a wavelength of 260 nm. The genomic DNA of phages vB_SenM-1, vB_SenM-2, and vB_SenS-3 were sequenced by the Genomed company with next generation sequencing (NGS) and MiSeq (Illumina) genome sequencing. Sequences of genomes were deposited in the GenBank database, and accession numbers for genomes of vB_SenM-1, vB_SenM-2, and vB_SenS-3 are MT012730, KX171211, and MT004791, respectively.

### 4.14. Annotation and Bioinformatic Analysis of Phages vB_SenM-1 and vB_SenS-3

Putative open reading frames (ORFs) that may encode gene products were predicted by using RASTtk [55], backed up by prodigal incorporated in Prokka annotation suite [56]. To confirm the selection of correct start codon, the ribosome binding sites were analyzed 4–12 nucleotides upstream of the start codon. Putative functions of translated products were then verified by BLASTn analysis of the Nucleotide Collection (nr/nt) database, additionally utilizing the CDD database pinpointing conserved domains [57], pVOGs database where (97) *Salmonella* phage genomes were obtained (http://dmk-brain.ecn.uiowa.edu/pVOGs/, last accessed on 7 August, 2020), and HMMer Reference Proteomes database [58]. The CGView Comparison Tool [59] was used to create a map of virus genome with GC skew and GC content analyses. To determine phage termini, packaging mode and topology of genomes PhageTerm was employed [60].

Comparison of ORFs from relative phages was performed by using the EasyFig program (http://mjsull.github.io/Easyfig/files.html, last accessed on 16 August, 2020). Classification of phages’ lifestyle was conducted in PHACTS [61]. Phage-specific promoters and Rho-independent transcriptional terminators in viral DNA sequences were predicted by the Neural Network Promoter Prediction NNPP method (http://www.fruitfly.org/seq_tools/promoter.html, last accessed on 7 August, 2020) and the Arnold tool [62], respectively.

Salmonella phages’ (vB_SenM-1, vB_SenS-3) genomes were sequenced on Illumina platform MiSeq, with Whole Genome Shotgun (WGS) strategy. The library was prepared using NEBNext DNA Library Prep Master Mix Set for Illumina; selection was random, with read length ranging from 36 to 251 bp. Raw reads were deposited in SRA databases under BioProject ID: PRJNA655386.

### 4.15. Phylogenetic Analysis

To construct phylogenetic trees, the nucleotide sequences of genes coding for terminase large subunits (genetic marker for the order *Caudovirales*) of vB_SenM-1, vB_SenM-2, and vB_SenS-3 viruses were compared with the sequences of other reference bacteriophages within the order *Caudovirales* that were deposited in the NCBI database. DNA sequences were aligned and adjusted by eye using Seaview [63]. The jModelTest v. 2.1.1 (Phylogenomics Lab, University of Vigo, Vigo, Spain) [64] was used in order to choose the best-fitting evolutionary model by the Akaike information criterion. All matrices were analyzed using PAUP (Phylogenetic Analysis Using Parsimony and Other Methods) version 4.0a [65]. The optimality criterion was set to distance using the neighbor-joining algorithm (NJ). The robustness of the tree topology was assessed by bootstrap analyses based on 1000 replicates.

## Figures and Tables

**Table 1:** Host range of phages vB_SenM-1, vB_SenM-2, and vB_SenS-3.

Bacterial Strain		Sensitivity to Phage ^c^		
*Salmonella enterica* Serotype ^a^	Source and Identification Number ^b^	vB_SenM-1	vB_SenM-2	vB_SenS-3
Heidelberg	DMB UG collection; no. UGSA2	100%	100%	100%
Panama	DMB UG collection; no. UGSA3	97.82% ± 3.28%	93.23% ± 4.21%	95.12% ± 6.16%
Reading	DMB UG collection; no. UGSA4	<0.01%	<0.01%	15.21% ± 1.11%
London	DMB UG collection; no. UGSA5	<0.01%	<0.01%	1.25% ± 0.09%
Anatum	NSC, no. 78	28.11% ± 3.16%	95.12% ± 4.11%	<0.01%
Typhimurium *	NSC, no. 12	67.13% ± 7.28%	98.20% ± 4.77%	95.13% ± 2.22%
Typhimurium	NSC, no. 13	23.28% ± 1.70%	22.40% ± 3.41%	98.61% ± 1.17%
Stanley *	NSC, no. 15	0.80% ± 0.06%	97.28% ± 2.16%	0.63% ± 0.11%
Heidelberg	NSC, no. 16	27.91% ± 8.09%	98.66% ± 1.06%	100% ± 1.03%
Cholerasuis	NSC, no. 34	17.22% ± 0.47%	97.81% ± 3.22%	99.82% ± 7.57%
Cholerasuis	NSC, no. 39	4.80% ± 0.88%	89.16% ± 7.22%	93.27% ± 4.21%
Cholerasuis var kunznedorf	NSC, no. 37	88.46% ± 2.61%	80.52% ± 7.36%	93.17% ± 4.16%
Cholerasuis	NSC, no. 1439	<0.01%	<0.01%	9.11% ± 0.76%
Virchow	NSC, no. 41	<0.01%	<0.01%	<0.01%
Newport	NSC, no. 50	17.26% ± 2.16%	10.08% ± 3.25%	<0.01%
Newport	NSC, no. 51	<0.01%	<0.01%	98.26% ± 3.89%
Enteritidis *	NSC, no. 64	88.87% ± 6.12%	95.18% ± 2.16%	98.26% ± 3.15%
Enteritidis *	NSC, no. 1392	66.21% ± 4.15%	81.22% ± 4.33%	86.36% ± 2.53%
Dublin	NSC, no. 65	55.21% ± 1.17%	88.25% ± 3.21%	97.16% ± 5.21%
Gallinarum	NSC, no. 74	63.55% ± 2.11%	91.66% ± 5.11%	89.21% ± 6.10%
Seftenberg	NSC, no. 87	<0.01%	<0.01%	94.66% ± 2.22%
Infantis *	NSC, no. 155	10.71% ± 1.06%	<0.01%	6.15% ± 0.91%
Bovismorbificans *	NSC, no. 300	<0.01%	<0.01%	94.27% ± 2.31%
Saintpaul	NSC, no. 435	63.28% ± 4.95%	96.21% ± 5.13%	89.92% ± 2.89%
Kentucky *	NSC, no. 1368	51.22% ± 7.33%	49.97% ± 3.47%	24.25% ± 2.97%
Agona *	NSC, no. 1408	29.18% ± 3.15%	98.36% ± 3.67%	95.22% ± 5.11%
Hadar *	NSC, no. 1784	<0.01%	<0.01%	7.66%

^a^ Resistance to the following antibiotics was tested using minimal inhibitory concentration (MIC) and disc diffusion tests: tetracycline, rifampicin, ampicillin, chloramphenicol, colistin, enrofloxacin, and kanamycin. The resistance/susceptibility was determined on the basis of EUCAST guidelines. Investigated strains were susceptible to all tested antibiotics, with the exception of colistin for strains indicated by asterisks. ^b^ Abbreviations: NSC, National *Salmonella* Centre, Poland; DMB UG, Department of Molecular Biology of University of Gdansk, Poland. ^c^ Phage titer on *S.* Heidelberg UGSA2 was used as a reference value (100%). Mean values from three independent experiments are shown with SD.

**Table 2:** Yield of progeny of phages vB_SenM-1, vB_SenM-2, and vB_SenS-3 per infected cell at different temperatures and on different *S. enterica* strains. Mean values from three independent experiments are shown with SD.

Phage	*S. enterica* Strain		Yield of Phage Progeny (PFU/Cell)		
	Serotype	No.	Temperature		
			25 °C	37 °C	42 °C
vB_SenM-1	Typhimurium	12	275.34 ± 30.27	58.31 ± 0.06	21.31 ± 5.56
		13	375.34 ± 75.85	206.20 ± 68.51	22.20 ± 3.06
	Enteritidis	64	13.35 ± 3.05	139.40 ± 3.55	105.48 ± 20.06
		1392	18.08 ± 6.98	499.22 ± 7.16	60.78 ± 18.37
vB_SenM-2	Typhimurium	12	109.70 ± 15.98	131.24 ± 17.88	12.29 ± 2.40
		13	12.76 ± 3.01	85.07 ± 4.91	59.06 ± 7.80
	Enteritidis	64	23.75 ± 5.34	229.79 ± 54.27	174.59 ± 10.06
		1392	153.94 ± 34.07	283.73 ± 71.35	2.41 ± 0.35
vB_SenS-3	Typhimurium	12	50.34 ± 18.98	136.21 ± 16.63	3.12 ± 0.91
		13	1.18 ± 0.21	77.15 ± 12.80	2.23 ± 0.49
	Enteritidis	64	28.75 ± 7.35	156.45 ± 31.15	7.40 ± 1.01
		1392	31.44 ± 19.17	117.38 ± 21.38	2.91 ± 1.57

**Table 3:** Sensitivity of phages vB_SenM-1, vB_SenM-2, and vB_SenS-3 to different pH levels and temperatures during 1-h incubation. pH = 7.0 was used as control value (100%).

Phage	Temperature (°C)		Phage Survival (%)					
			pH					
		7.0	1.8	2.0	2.2	2.5	2.8	3.0
vB_SenM-1	25 °C	100% ± 0.59%	<0.01%	<0.01%	<0.01%	3.23% ± 0.03%	4.83% ± 0.01%	4.51% ± 0.09%
	37 °C	100%	<0.01%	<0.01%	<0.01%	<0.01%	2.22% ± 0.01%	5.71% ± 0.12%
	42 °C	95.12% ± 2.12%	<0.01%	<0.01%	<0.01%	<0.01%	<0.01%	3.26% ± 0.01%
vB_SenM-2	25 °C	98.66% ± 1.1%	<0.01%	<0.01%	3.14% ± 0.02%	2.23% ± 0.06%	3.62% ± 0.11%	12.83% ± 1.13%
	37 °C	100%	<0.01%	<0.01%	2.13% ± 0.12%	2.7% ± 0.03%	2.54% ± 0.06%	18.27% ± 3.16%
	42 °C	86.93% ± 6.27%	<0.01%	<0.01%	<0.01%	1.7% ± 0.01%	2.13% ± 0.12%	6.32% ± 0.42%
vB_SenS-3	25 °C	100% ± 2.19%	<0.01%	<0.01%	1.92% ± 0.24%	2.43% ± 0.33%	2.32% ± 0.07%	7.93% ± 0.04%
	37 °C	100%	<0.01%	<0.01%	<0.01%	1.71% ± 0.04%	4.31% ± 0.24%	6.15% ± 1.13%
	42 °C	81.26% ± 3.44%	<0.01%	<0.01%	<0.01%	<0.01%	12.14% ± 3.16%	13.33% ± 0.72%

## Supplementary Materials

Supplementary materials can be found at https://www.mdpi.com/1422-0067/21/17/6152/s1. Table S1. Genome annotations of phage vB_SenM-1. Table S2. Genome annotations of phage vB_SenS-3. Figure S1. Electron micrographs of phages vB_SenM-1 (A), vB_SenM-2 (B) and vB_SenS-3 (C). Scale bars represent 50 nM. Figure S2. Changes in PFU/ml values of phages vB_SenM-1 (closed circles), vB_SenM-2 (open squares) and vB_SenS-3 (open triangles) after infection of bacterial cultures of S. Typhimurium (A, B) and S. Enteritidis (C, D) at m.o.i=1. Mean values from three independent experiments are shown, with error bars representing SD. Figure S3. Changes in PFU/ml values of phages vB_SenM-1 (closed circles), vB_SenM-2 (open squares) and vB_SenS-3 (open triangles) after infection of bacterial cultures of S. Typhimurium (A, B) and S. Enteritidis (C, D) at m.o.i=0.5. Mean values from three independent experiments are shown, with error bars representing SD. Figure S4. Changes in PFU/ml values of phages vB_SenM-1 (closed circles), vB_SenM-2 (open squares) and vB_SenS-3 (open triangles) after infection of bacterial cultures of S. Typhimurium (A, B) and S. Enteritidis (C, D) at m.o.i=0.1. Mean values from three independent experiments are shown, with error bars representing SD. Figure S5. Changes in CFU/ml values of cultures of S. Typhimurium (A, B) and S. Enteritidis (C, D) infected with phages vB_SenM-1 (closed circles), vB_SenM-2 (open squares) and vB_SenS-3 (closed triangles) at m.o.i=0.5, compared with uninfected control (closed squares). Mean values from three independent experiments shown, with error bars representing SD. Figure S6. Changes in CFU/ml values of bacterial cultures of S. Typhimurium (A, B) and S. Enteritidis (C, D) infected with phages vB_SenM-1 (closed circles), vB_SenM-2 (open squares) and vB_SenS-3 (closed triangles) at m.o.i=0.1, compared with uninfected control (closed squares). Mean values from three independent experiments are shown, with error bars representing SD. Figure S7. Lysis profile of S. Typhimurium (A, B) and S. Enteritidis (C, D) infected with phages vB_SenM-1 (closed circles), vB_SenM-2 (open squares) and vB_SenS-3 (open triangles) at m.o.i=1 compared with uninfected control (closed squares). Mean values from three independent experiments are shown, with error bars representing SD. Figure S8. Lysis profile of S. Typhimurium (A, B) and S. Enteritidis (C, D) infected with phages vB_SenM-1 (closed circles), vB_SenM-2 (open squares) and vB_SenS-3 (open triangles) at m.o.i=0.5 compared with uninfected control (closed squares). Mean values from three independent experiments are shown, with error bars representing SD. Figure S9. Lysis profile of S. Typhimurium (A, B) and S. Enteritidis (C, D) infected with phages vB_SenM-1 (closed circles), vB_SenM-2 (open squares) and vB_SenS-3 (open triangles) at m.o.i=0.1 compared with uninfected control (closed squares). Mean values from three independent experiments are shown, with error bars representing SD.
