# Supplementary material for: Characteristics of a Series of Three Bacteriophages Infecting Salmonella enterica Strains
Source: Int J Mol Sci. 2020 Aug 26;21(17):6152. doi: 10.3390/ijms21176152 (PMC7503781; doi:10.3390/ijms21176152)
Supplement: Supplementary file 1 [file ijms-21-06152-s001.zip › SEN-KKK-Legends to Supplementary Figures-R1.pdf]

Supplementary Material

Legends to Supplementary Figures

Figure S1. Electron micrographs of phages vB_SenM-1 (A), vB_SenM-2 (B) and vB_SenS-3 (C). Scale bars represent 50 nM.

Figure S2. Changes in PFU/ml values of phages vB_SenM-1 (closed circles), vB_SenM-2 (open squares) and vB_SenS-3 (open triangles) after infection of bacterial cultures of *S.* Typhimurium (A, B) and *S.* Enteritidis (C, D) at m.o.i=1. Mean values from three independent experiments are shown, with error bars representing SD.

Figure S3. Changes in PFU/ml values of phages vB_SenM-1 (closed circles), vB_SenM-2 (open squares) and vB_SenS-3 (open triangles) after infection of bacterial cultures of *S.* Typhimurium (A, B) and *S.* Enteritidis (C, D) at m.o.i=0.5. Mean values from three independent experiments are shown, with error bars representing SD.

Figure S4. Changes in PFU/ml values of phages vB_SenM-1 (closed circles), vB_SenM-2 (open squares) and vB_SenS-3 (open triangles) after infection of bacterial cultures of *S.* Typhimurium (A, B) and *S.* Enteritidis (C, D) at m.o.i=0.1. Mean values from three independent experiments are shown, with error bars representing SD.

Figure S5. Changes in CFU/ml values of cultures of *S.* Typhimurium (A, B) and *S.* Enteritidis (C, D) infected with phages vB_SenM-1 (closed circles), vB_SenM-2 (open squares) and vB_SenS-3 (closed triangles) at m.o.i=0.5, compared with uninfected control (closed squares). Mean values from three independent experiments shown, with error bars representing SD.

Figure S6. Changes in CFU/ml values of bacterial cultures of *S.* Typhimurium (A, B) and *S.* Enteritidis (C, D) infected with phages vB_SenM-1 (closed circles), vB_SenM-2 (open squares) and vB_SenS-3 (closed triangles) at m.o.i=0.1, compared with uninfected control (closed squares). Mean values from three independent experiments are shown, with error bars representing SD.

Figure S7. Lysis profile of *S.* Typhimurium (A, B) and *S.* Enteritidis (C, D) infected with phages vB_SenM-1 (closed circles), vB_SenM-2 (open squares) and vB_SenS-3 (open triangles) at m.o.i=1 compared with uninfected control (closed squares). Mean values from three independent experiments are shown, with error bars representing SD.

Figure S8. Lysis profile of *S.* Typhimurium (A, B) and *S.* Enteritidis (C, D) infected with phages vB_SenM-1 (closed circles), vB_SenM-2 (open squares) and vB_SenS-3 (open triangles) at m.o.i=0.5 compared with uninfected control (closed squares). Mean values from three independent experiments are shown, with error bars representing SD.

Figure S9. Lysis profile of *S.* Typhimurium (A, B) and *S.* Enteritidis (C, D) infected with phages vB_SenM-1 (closed circles), vB_SenM-2 (open squares) and vB_SenS-3 (open triangles) at m.o.i=0.1 compared with uninfected control (closed squares). Mean values from three independent experiments are shown, with error bars representing SD.
