# Supplementary material for: Characteristics of a Series of Three Bacteriophages Infecting Salmonella enterica Strains
Source: Int J Mol Sci. 2020 Aug 26;21(17):6152. doi: 10.3390/ijms21176152 (PMC7503781; doi:10.3390/ijms21176152)
Supplement: Supplementary file 1 [file ijms-21-06152-s001.zip › SEN-KKK-Table-S1-R1.pdf]

Table S1. Genome annotations of phage vB_SenM-1

Locus_tag	Start	Stop	Strand	Product
vB_SenM-1_01	634	2	-	Similar to hypothetical protein
vB_SenM-1_02	1549	665	-	Thymidylate synthase (EC 2.1.1.45)
vB_SenM-1_03	2142	1549	-	Cof hydrolase
vB_SenM-1_04	2527	2129	-	hypothetical protein
vB_SenM-1_05	2679	2590	-	hypothetical protein
vB_SenM-1_06	2944	2822	-	hypothetical protein
vB_SenM-1_07	3102	3010	-	hypothetical protein
vB_SenM-1_08	3191	3099	-	hypothetical protein
vB_SenM-1_09	3729	3202	-	hypothetical protein
vB_SenM-1_10	3997	3776	-	hypothetical protein
vB_SenM-1_11	5654	3984	-	superfamily II helicase
vB_SenM-1_12	6591	5713	-	hypothetical protein
vB_SenM-1_13	7060	6575	-	deoxycytidylate deaminase
vB_SenM-1_14	7667	7122	-	hypothetical protein
vB_SenM-1_15	8659	7775	-	ATP-binding protein
vB_SenM-1_16	8889	8659	-	hypothetical protein
vB_SenM-1_17	9396	8950	-	hypothetical protein
vB_SenM-1_18	10347	9445	-	hypothetical protein
vB_SenM-1_19	10752	10462	-	hypothetical protein
vB_SenM-1_20	10855	10754	-	hypothetical protein
vB_SenM-1_21	11177	10830	-	hypothetical protein
vB_SenM-1_22	11671	11222	-	hypothetical protein
vB_SenM-1_23	11728	11934	+	hypothetical protein

vB_SenM-1_24	12286	11945	-	hypothetical protein
vB_SenM-1_25	12804	12283	-	hypothetical protein
vB_SenM-1_26	12874	13206	+	hypothetical protein
vB_SenM-1_27	13181	15715	+	DNA primase/helicase
vB_SenM-1_28	15876	16040	+	hypothetical protein
vB_SenM-1_29	16042	16146	+	hypothetical protein
vB_SenM-1_30	16213	16644	+	hypothetical protein
vB_SenM-1_31	16681	16779	+	hypothetical protein
vB_SenM-1_32	16802	17083	+	hypothetical protein
vB_SenM-1_33	17225	17058	-	hypothetical protein
vB_SenM-1_34	17362	17237	-	hypothetical protein
vB_SenM-1_35	17723	17424	-	hypothetical protein
vB_SenM-1_36	17981	17781	-	hypothetical protein
vB_SenM-1_37	18370	17981	-	hypothetical protein
vB_SenM-1_38	18612	18439	-	hypothetical protein
vB_SenM-1_39	18866	18609	-	hypothetical protein
vB_SenM-1_40	19030	18872	-	hypothetical protein
vB_SenM-1_41	19665	19096	-	hypothetical protein
vB_SenM-1_42	19898	19761	-	hypothetical protein
vB_SenM-1_43	20216	20115	-	hypothetical protein
vB_SenM-1_44	20304	20200	-	hypothetical protein
vB_SenM-1_45	20461	20601	+	hypothetical protein
vB_SenM-1_46	20586	20705	+	hypothetical protein
vB_SenM-1_47	20748	21230	+	hypothetical protein

vB_SenM-1_48	21244	21897	+	dihydrofolate reductase
vB_SenM-1_49	21894	22400	+	guanylate kinase
vB_SenM-1_50	22397	23107	+	hypothetical protein
vB_SenM-1_51	23107	23397	+	hypothetical protein
vB_SenM-1_52	23575	23865	+	hypothetical protein
vB_SenM-1_53	23869	25317	+	terminase large subunit
vB_SenM-1_54	25319	26881	+	portal protein
vB_SenM-1_55	26921	27022	+	hypothetical protein
vB_SenM-1_56	27059	27772	+	scaffold protein
vB_SenM-1_57	28864	27779	-	hypothetical protein
vB_SenM-1_58	28858	29358	+	hypothetical protein
vB_SenM-1_59	29358	29846	+	hypothetical protein
vB_SenM-1_60	29846	30223	+	hypothetical protein
vB_SenM-1_61	30147	30674	+	hypothetical protein
vB_SenM-1_62	30674	31297	+	putative tail fibers protein
vB_SenM-1_63	31297	34254	+	tail formation protein GpI
vB_SenM-1_64	34322	34960	+	hypothetical protein
vB_SenM-1_65	34972	36861	+	major tail protein
vB_SenM-1_66	36948	38087	+	hypothetical protein
vB_SenM-1_67	38099	38530	+	hypothetical protein
vB_SenM-1_68	38549	38977	+	hypothetical protein
vB_SenM-1_69	39046	39159	+	hypothetical protein
vB_SenM-1_70	39140	40801	+	tail tape measure protein
vB_SenM-1_71	40798	41694	+	hypothetical protein

vB_SenM-1_72	41737	41844	+	hypothetical protein
vB_SenM-1_73	42007	42987	+	hypothetical protein
vB_SenM-1_74	42987	43634	+	translation initiation factor IF-2
vB_SenM-1_75	43645	44016	+	hypothetical protein
vB_SenM-1_76	44016	45188	+	hypothetical protein
vB_SenM-1_77	45175	45834	+	hypothetical protein
vB_SenM-1_78	45821	47161	+	tail fiber protein
vB_SenM-1_79	47170	47712	+	putative tail fibers protein
vB_SenM-1_80	47716	48219	+	putative tail fibers protein
vB_SenM-1_81	48322	48582	+	hypothetical protein
vB_SenM-1_82	48626	49102	+	lysozyme R (EC 3.2.1.17)
vB_SenM-1_83	49081	49410	+	hypothetical protein
vB_SenM-1_84	49355	49612	+	hypothetical protein
vB_SenM-1_85	49852	49628	-	hypothetical protein
vB_SenM-1_86	50127	49852	-	hypothetical protein
vB_SenM-1_87	52110	50143	-	DNA polymerase I (EC 2.7.7.7)
vB_SenM-1_88	52427	52110	-	DNA polymerase beta subunit
