# Supplementary material for: Characteristics of a Series of Three Bacteriophages Infecting Salmonella enterica Strains
Source: Int J Mol Sci. 2020 Aug 26;21(17):6152. doi: 10.3390/ijms21176152 (PMC7503781; doi:10.3390/ijms21176152)
Supplement: Supplementary file 1 [file ijms-21-06152-s001.zip › SEN-KKK-Table-S2-R1.pdf]

Table S2. Genome annotations of phage vB_SenS-3

**Locus tag**	**Start**	**Stop**	**Strand**	**Product**
vB_SenS-3_01	844	122	-	deoxynucleoside-5'-monophosphatase
vB_SenS-3_02	1319	924	-	hypothetical protein
vB_SenS-3_03	1604	1362	-	hypothetical protein
vB_SenS-3_04	3322	1658	-	putative A1 protein
vB_SenS-3_05	3658	3431	-	hypothetical protein
vB_SenS-3_06	4124	3708	-	A2 protein
vB_SenS-3_07	4662	4411	-	hypothetical protein
vB_SenS-3_08	4681	4782	+	hypothetical protein
vB_SenS-3_09	4910	5056	+	hypothetical protein
vB_SenS-3_10	5121	5246	+	hypothetical protein
vB_SenS-3_11	5367	5480	+	hypothetical protein
vB_SenS-3_12	6119	5874	-	hypothetical protein
vB_SenS-3_13	6147	7148	+	hypothetical protein
vB_SenS-3_14	7268	7417	+	putative membrane protein
vB_SenS-3_15	7420	7632	+	hypothetical protein
vB_SenS-3_16	7634	7978	+	hypothetical protein
vB_SenS-3_17	7975	8160	+	hypothetical protein
vB_SenS-3_18	8153	8293	+	hypothetical protein
vB_SenS-3_19	8284	8523	+	hypothetical protein
vB_SenS-3_20	8676	8452	-	hypothetical protein
vB_SenS-3_21	8895	9056	+	hypothetical protein
vB_SenS-3_22	9103	9276	+	hypothetical protein
vB_SenS-3_23	9357	9563	+	hypothetical protein

vB_SenS-3_24	9868	9966	+	hypothetical protein
vB_SenS-3_25	11005	10124	-	hypothetical protein
vB_SenS-3_26	11432	11082	-	hypothetical protein
vB_SenS-3_27	12037	11432	-	hypothetical protein
vB_SenS-3_28	12222	12037	-	putative membrane protein
vB_SenS-3_29	12455	12222	-	hypothetical protein
vB_SenS-3_30	12741	12457	-	hypothetical protein
vB_SenS-3_31	13183	12665	-	hypothetical protein
vB_SenS-3_32	13611	13228	-	capsid and scaffold protein
vB_SenS-3_33	14020	13553	-	hypothetical protein
vB_SenS-3_34	14217	14017	-	putative membrane protein
vB_SenS-3_35	14320	14201	-	hypothetical protein
vB_SenS-3_36	14649	14317	-	hypothetical protein
vB_SenS-3_37	14884	14639	-	putative membrane protein
vB_SenS-3_38	15162	14881	-	putative membrane protein
vB_SenS-3_39	15320	15162	-	hypothetical protein
vB_SenS-3_40	15570	15310	-	hypothetical protein
vB_SenS-3_41	16076	15645	-	hypothetical protein
vB_SenS-3_42	16246	16145	-	hypothetical protein
vB_SenS-3_43	16706	16239	-	HNH homing endonuclease
vB_SenS-3_44	17221	16703	-	phosphoesterase
vB_SenS-3_45	17589	17221	-	hypothetical protein
vB_SenS-3_46	18452	17589	-	putative serine/threonine protein phosphatase
vB_SenS-3_47	18845	18555	-	thioredoxin

vB_SenS-3_48	19248	18838	-	hypothetical protein
vB_SenS-3_49	19740	19324	-	putative membrane protein
vB_SenS-3_50	20229	19816	-	endolysin
vB_SenS-3_51	20882	20226	-	holin
vB_SenS-3_52	21638	21039	-	ATP-dependent Clp protease proteolytic subunit
vB_SenS-3_53	22403	21651	-	dNMP kinase
vB_SenS-3_54	22756	22403	-	hypothetical protein
vB_SenS-3_55	23136	22687	-	i-spanin
vB_SenS-3_56	23791	23093	-	hypothetical protein
vB_SenS-3_57	23908	23813	-	hypothetical protein
vB_SenS-3_58	24284	23937	-	putative membrane protein
vB_SenS-3_59	24685	24401	-	putative membrane protein
vB_SenS-3_60	24978	24682	-	hypothetical protein
vB_SenS-3_61	25375	24932	-	hypothetical protein
vB_SenS-3_62	25643	25344	-	hypothetical protein
vB_SenS-3_63	25915	25643	-	hypothetical protein
vB_SenS-3_64	26387	25992	-	hypothetical protein
vB_SenS-3_65	26631	26446	-	hypothetical protein
vB_SenS-3_66	26857	26696	-	hypothetical protein
vB_SenS-3_67	27077	26916	-	hypothetical protein
vB_SenS-3_68	27445	27077	-	2-ketobutyrate formate-lyase (EC 2.3.1.-) @ Pyruvate formate-lyase (EC 2.3.1.54)
vB_SenS-3_69	27581	27423	-	hypothetical protein
vB_SenS-3_70	27713	27582	-	hypothetical protein

vB_SenS-3_71	27879	27682	-	hypothetical protein
vB_SenS-3_72	28266	28060	-	hypothetical protein
vB_SenS-3_73	28559	28266	-	hypothetical protein
vB_SenS-3_74	28687	28580	-	hypothetical protein
vB_SenS-3_75	28883	28719	-	hypothetical protein
vB_SenS-3_76	29097	28876	-	hypothetical protein
vB_SenS-3_77	29337	29179	-	hypothetical protein
vB_SenS-3_78	29560	29360	-	hypothetical protein
vB_SenS-3_79	29744	29631	-	hypothetical protein
vB_SenS-3_80	29810	29914	+	hypothetical protein
vB_SenS-3_81	29922	30080	+	hypothetical protein
vB_SenS-3_82	30384	30142	-	hypothetical protein
vB_SenS-3_83	30656	30480	-	hypothetical protein
vB_SenS-3_84	30747	30634	-	hypothetical protein
vB_SenS-3_85	30979	30761	-	hypothetical protein
vB_SenS-3_86	31059	30967	-	hypothetical protein
vB_SenS-3_87	31214	31065	-	hypothetical protein
vB_SenS-3_88	31795	31442	-	hypothetical protein
vB_SenS-3_89	32146	31982	-	hypothetical protein
vB_SenS-3_90	32207	32380	+	hypothetical protein
vB_SenS-3_91	32413	32523	+	hypothetical protein
vB_SenS-3_92	32687	32562	-	hypothetical protein
vB_SenS-3_93	32890	32750	-	hypothetical protein
vB_SenS-3_94	33646	33065	-	homing endonuclease

vB_SenS-3_95	33835	33650	-	hypothetical protein
vB_SenS-3_96	34051	33917	-	hypothetical protein
vB_SenS-3_97	34243	34133	-	hypothetical protein
vB_SenS-3_98	34440	34252	-	hypothetical protein
vB_SenS-3_99	34762	34490	-	hypothetical protein
vB_SenS-3_100	34782	34913	+	hypothetical protein
vB_SenS-3_101	35138	35001	-	hypothetical protein
vB_SenS-3_102	35571	35296	-	hypothetical protein
vB_SenS-3_103	35933	35661	-	hypothetical protein
vB_SenS-3_104	36138	36046	-	hypothetical protein
vB_SenS-3_105	36367	36215	-	hypothetical protein
vB_SenS-3_106	36936	36427	-	H-N-H-endonuclease F-TflVI
vB_SenS-3_107	37303	37007	-	hypothetical protein
vB_SenS-3_108	38087	37410	-	PnuC-like ribosyl nicotinamide transporter
vB_SenS-3_109	39195	38089	-	ribosylnicotinamide kinase
vB_SenS-3_110	39312	39404	+	hypothetical protein
vB_SenS-3_111	40352	39408	-	putative SPFH domain-containing protein
vB_SenS-3_112	40564	40364	-	hypothetical protein
vB_SenS-3_113	41183	40674	-	hypothetical protein
vB_SenS-3_114	41399	41232	-	hypothetical protein
vB_SenS-3_115	41728	41405	-	hypothetical protein
vB_SenS-3_116	42296	41814	-	Phage-associated homing endonuclease
vB_SenS-3_117	42783	42340	-	recombination related exonuclease
vB_SenS-3_118	42953	42783	-	hypothetical protein

vB_SenS-3_119	43472	43023	-	cell wall hydrolase
vB_SenS-3_120	43795	43478	-	hypothetical protein
vB_SenS-3_121	44131	44024	-	hypothetical protein
vB_SenS-3_122	44883	44245	-	Phage tail fiber protein
vB_SenS-3_123	45119	44937	-	hypothetical protein
vB_SenS-3_124	45891	45190	-	Metallopeptidase phage-associated
vB_SenS-3_125	46134	45922	-	hypothetical protein
vB_SenS-3_126	46391	46176	-	tail tape measure protein
vB_SenS-3_127	46968	46453	-	hypothetical protein
vB_SenS-3_128	47330	47052	-	hypothetical protein
vB_SenS-3_129	47883	47407	-	Phage ribonuclease H (EC 3.1.26.4)
vB_SenS-3_130	48152	47883	-	hypothetical protein
vB_SenS-3_131	48406	48152	-	hypothetical protein
vB_SenS-3_132	48852	48499	-	hypothetical protein
vB_SenS-3_133	49710	48856	-	Thymidylate synthase (EC 2.1.1.45)
vB_SenS-3_134	50237	49707	-	Dihydrofolate reductase phage-associated
vB_SenS-3_135	51382	50237	-	Ribonucleotide reductase of class Ia (aerobic) beta subunit (EC 1.17.4.1)
vB_SenS-3_136	53880	51490	-	Ribonucleotide reductase of class Ia (aerobic) alpha subunit (EC 1.17.4.1)
vB_SenS-3_137	54186	53944	-	hypothetical protein
vB_SenS-3_138	54940	54188	-	Phage phosphate starvation-inducible protein PhoH (ACLAME 589)
vB_SenS-3_139	54957	55061	+	hypothetical protein
vB_SenS-3_140	55115	55258	+	hypothetical protein

vB_SenS-3_141	55296	57170	+	Ribonucleotide reductase of class III (anaerobic) large subunit (EC 1.17.4.2)
vB_SenS-3_142	57269	57550	+	hypothetical protein
vB_SenS-3_143	57560	57763	+	hypothetical protein
vB_SenS-3_144	57732	57926	+	hypothetical protein
vB_SenS-3_145	57926	58777	+	NAD-dependent protein deacetylase of SIR2 family
vB_SenS-3_146	58755	58937	+	hypothetical protein
vB_SenS-3_147	58918	59103	+	hypothetical protein
vB_SenS-3_148	59057	59596	+	hypothetical protein
vB_SenS-3_149	59635	60027	+	hypothetical protein
vB_SenS-3_150	60037	60432	+	hypothetical protein
vB_SenS-3_151	60467	60568	+	hypothetical protein
vB_SenS-3_152	60829	60698	-	hypothetical protein
vB_SenS-3_153	60926	63838	+	DNA primase C
vB_SenS-3_154	63822	64055	+	hypothetical protein
vB_SenS-3_155	64124	64828	+	D2 protein
vB_SenS-3_156	64821	65075	+	hypothetical protein
vB_SenS-3_157	65183	65593	+	putative D3 protein
vB_SenS-3_158	65630	65926	+	hypothetical protein
vB_SenS-3_159	65977	66285	+	transcriptional regulator protein
vB_SenS-3_160	66371	66469	+	hypothetical protein
vB_SenS-3_161	66561	67535	+	D DNA ligase phage-associated
vB_SenS-3_162	67508	67630	+	hypothetical protein
vB_SenS-3_163	67738	68517	+	DNA ligase phage-associated
vB_SenS-3_164	68510	69277	+	D5 protein

vB_SenS-3_165	69309	70832	+	putative DNA helicase
vB_SenS-3_166	70829	71719	+	DNA primase/helicase
vB_SenS-3_167	71782	74349	+	DNA polymerase phage-associated
vB_SenS-3_168	74342	74839	+	hypothetical protein
vB_SenS-3_169	74836	76182	+	Phage DNA helicase
vB_SenS-3_170	76184	76714	+	Phage-associated homing endonuclease
vB_SenS-3_171	76880	77242	+	hypothetical protein
vB_SenS-3_172	77235	78008	+	hypothetical protein
vB_SenS-3_173	78048	79025	+	Phage-associated recombinase
vB_SenS-3_174	79006	80844	+	Phage recombination related exonuclease (EC 3.1.11.-)
vB_SenS-3_175	80848	81330	+	D14 protein
vB_SenS-3_176	81330	82205	+	Phage endonuclease
vB_SenS-3_177	82202	82648	+	Deoxyuridine 5'-triphosphate nucleotidohydrolase (EC 3.6.1.23)
vB_SenS-3_178	82711	82887	+	hypothetical protein
vB_SenS-3_179	83264	83055	-	hypothetical protein
vB_SenS-3_180	83609	83280	-	hypothetical protein
vB_SenS-3_181	86032	83765	-	L-shaped tail fiber
vB_SenS-3_182	86454	86032	-	lateral fiber collar protein
vB_SenS-3_183	88517	86460	-	tail fiber protein
vB_SenS-3_184	91367	88518	-	tail fiber protein
vB_SenS-3_185	91978	91364	-	distal tail protein
vB_SenS-3_186	95794	92087	-	tail fiber protein
vB_SenS-3_187	95897	95787	-	hypothetical protein

vB_SenS-3_188	96243	95875	-	putative tape measure chaperone
vB_SenS-3_189	96709	96305	-	putative tape measure chaperone
vB_SenS-3_190	97605	96706	-	tail fiber protein
vB_SenS-3_191	99019	97610	-	major tail protein
vB_SenS-3_192	99531	99046	-	hypothetical protein
vB_SenS-3_193	100302	99535	-	hypothetical protein
vB_SenS-3_194	100814	100302	-	head completion protein
vB_SenS-3_195	102250	100874	-	capsid and scaffold protein
vB_SenS-3_196	102900	102268	-	capsid and scaffold protein
vB_SenS-3_197	103386	102904	-	tail fiber protein
vB_SenS-3_198	104600	103383	-	portal (connector) protein
vB_SenS-3_199	105037	104600	-	putative nicking site-specific endonuclease
vB_SenS-3_200	106468	105152	-	terminase large subunit
vB_SenS-3_201	106950	106468	-	putative terminase small subunit
vB_SenS-3_202	108733	106961	-	receptor-binding tail protein
vB_SenS-3_203	108817	109083	+	putative receptor blocking protein
vB_SenS-3_204	109158	109469	+	hypothetical protein
vB_SenS-3_205	109542	109646	+	hypothetical protein
vB_SenS-3_206	109646	109840	+	hypothetical protein
vB_SenS-3_207	109833	110078	+	putative membrane protein
vB_SenS-3_208	110977	110243	-	deoxynucleoside-5'-monophosphatase
vB_SenS-3_209	111452	111057	-	hypothetical protein
vB_SenS-3_210	111737	111495	-	hypothetical protein
vB_SenS-3_211	113455	111791	-	putative A1 protein

vB_SenS-3_212	113791	113564	-	hypothetical protein
vB_SenS-3_213	114259	113843	-	A2 protein
vB_SenS-3_214	114797	114546	-	hypothetical protein
vB_SenS-3_215	114816	114917	+	hypothetical protein
vB_SenS-3_216	115045	115191	+	hypothetical protein
vB_SenS-3_217	115256	115381	+	hypothetical protein
vB_SenS-3_218	115502	115615	+	hypothetical protein
vB_SenS-3_219	116254	116009	-	hypothetical protein
vB_SenS-3_220	116282	117283	+	hypothetical protein
vB_SenS-3_221	117403	117552	+	putative membrane protein
vB_SenS-3_222	117555	117767	+	hypothetical protein
vB_SenS-3_223	117769	118113	+	hypothetical protein
vB_SenS-3_224	118110	118295	+	hypothetical protein
vB_SenS-3_225	118288	118428	+	hypothetical protein
vB_SenS-3_226	118419	118658	+	hypothetical protein
vB_SenS-3_227	118811	118587	-	hypothetical protein
vB_SenS-3_228	119030	119191	+	hypothetical protein
vB_SenS-3_229	119238	119411	+	hypothetical protein
vB_SenS-3_230	119492	119585	+	hypothetical protein
