# Supplementary figures and images for: Characteristics of a Series of Three Bacteriophages Infecting Salmonella enterica Strains
Source: Int J Mol Sci. 2020 Aug 26;21(17):6152. doi: 10.3390/ijms21176152 (PMC7503781; doi:10.3390/ijms21176152)

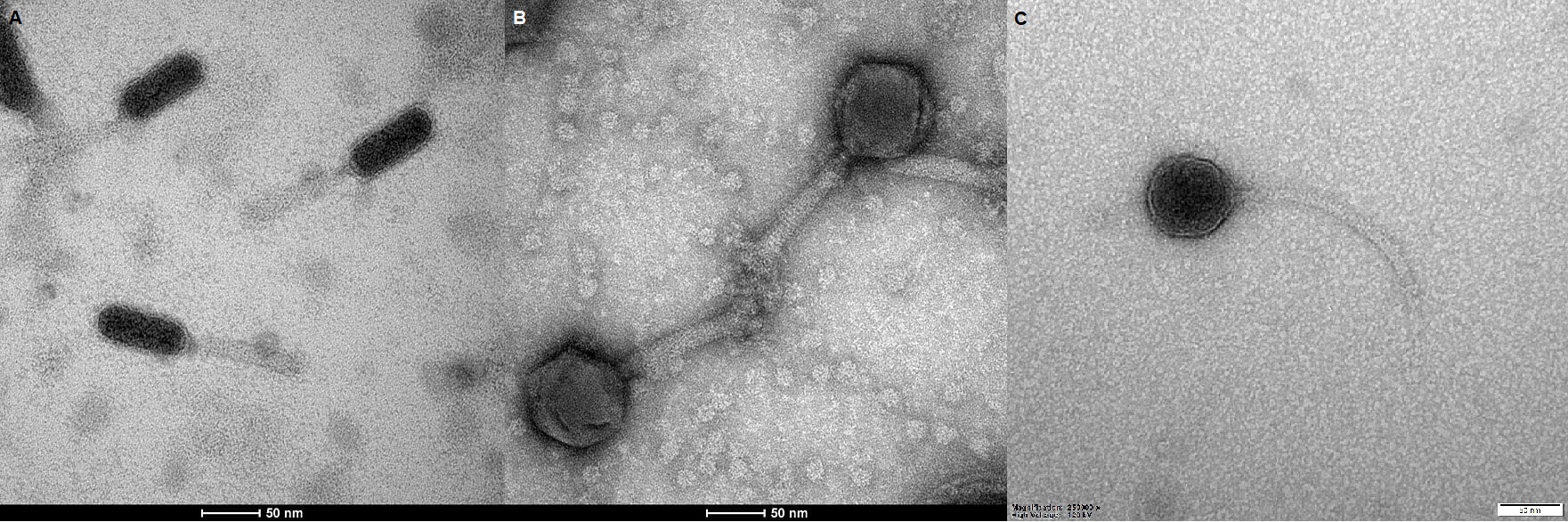

Supplement: Supplementary file 1 [file ijms-21-06152-s001.zip › SEN-KKK-Suppl-Figure S1-R1.jpg]

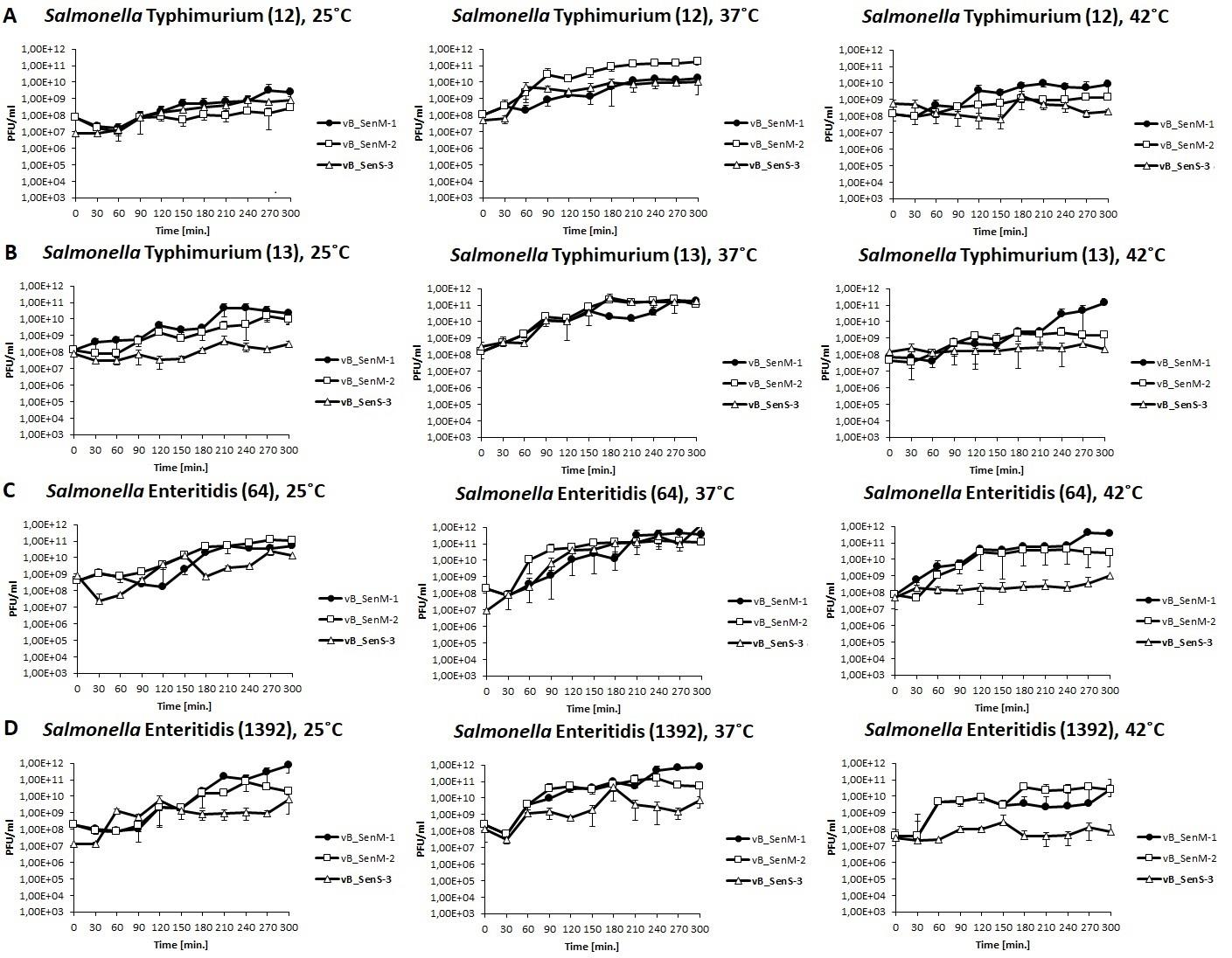

Supplement: Supplementary file 1 [file ijms-21-06152-s001.zip › SEN-KKK-Suppl-Figure S2-R1.jpg]

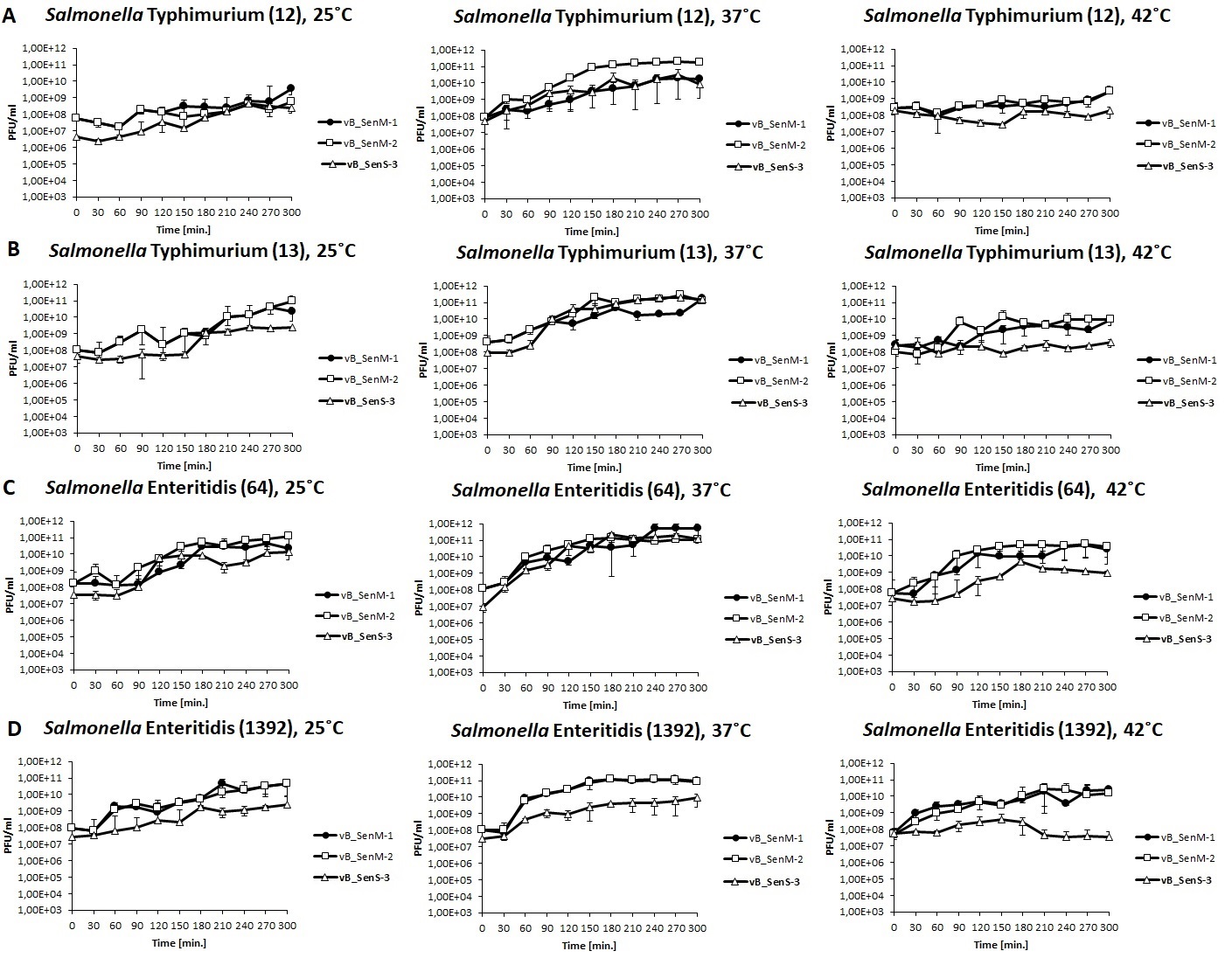

Supplement: Supplementary file 1 [file ijms-21-06152-s001.zip › SEN-KKK-Suppl-Figure S3-R1.jpg]

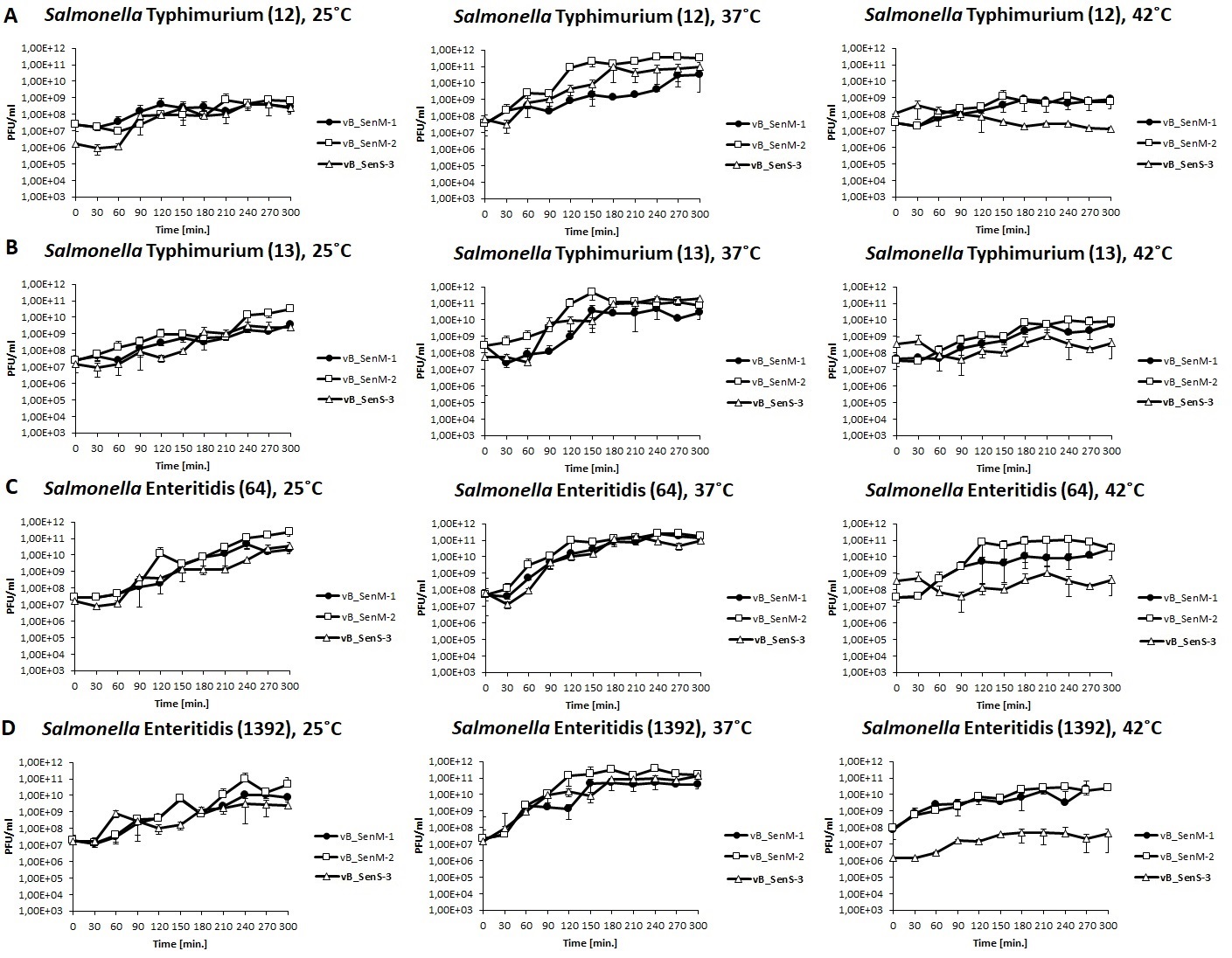

Supplement: Supplementary file 1 [file ijms-21-06152-s001.zip › SEN-KKK-Suppl-Figure S4-R1.jpg]

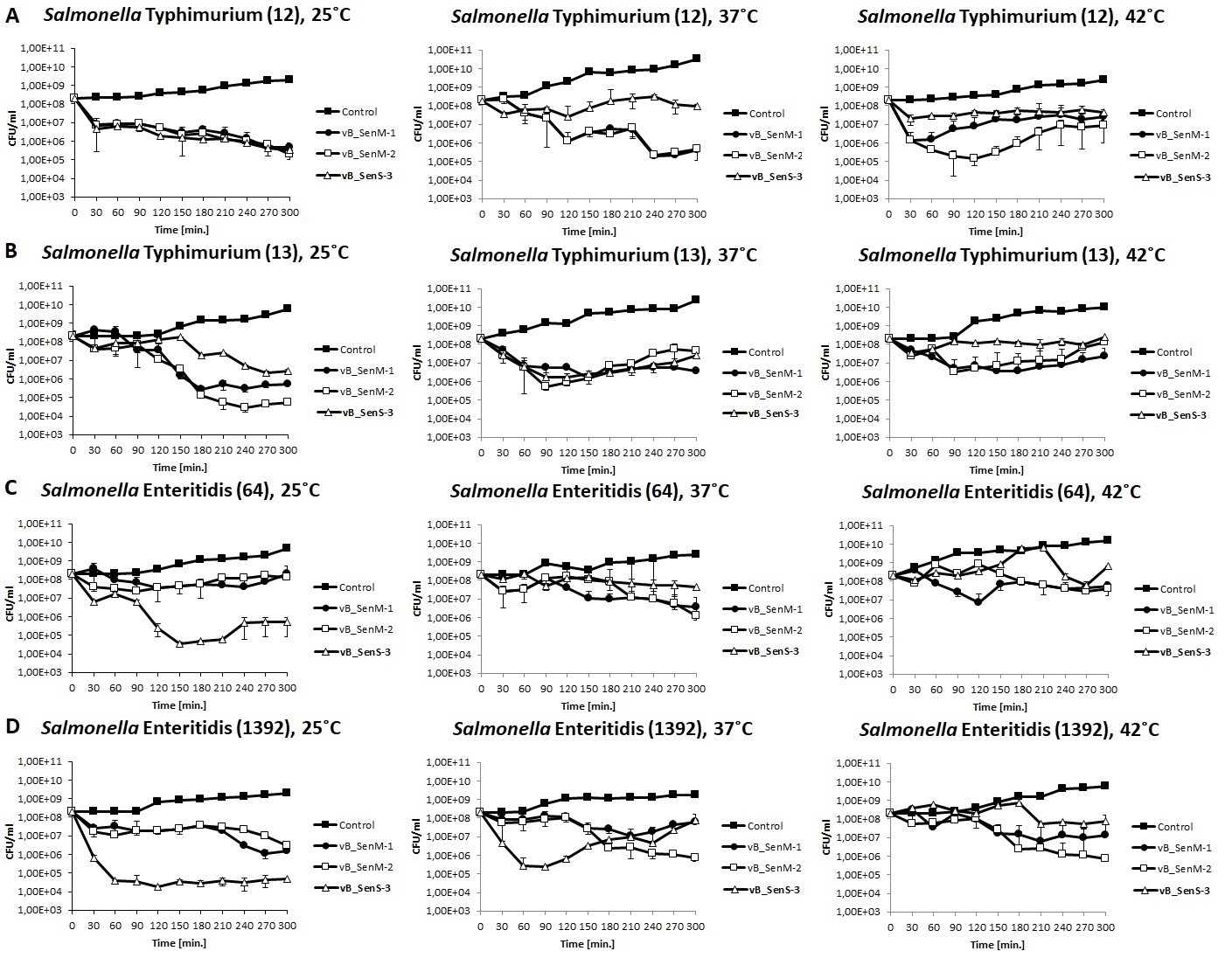

Supplement: Supplementary file 1 [file ijms-21-06152-s001.zip › SEN-KKK-Suppl-Figure S5-R1.jpg]

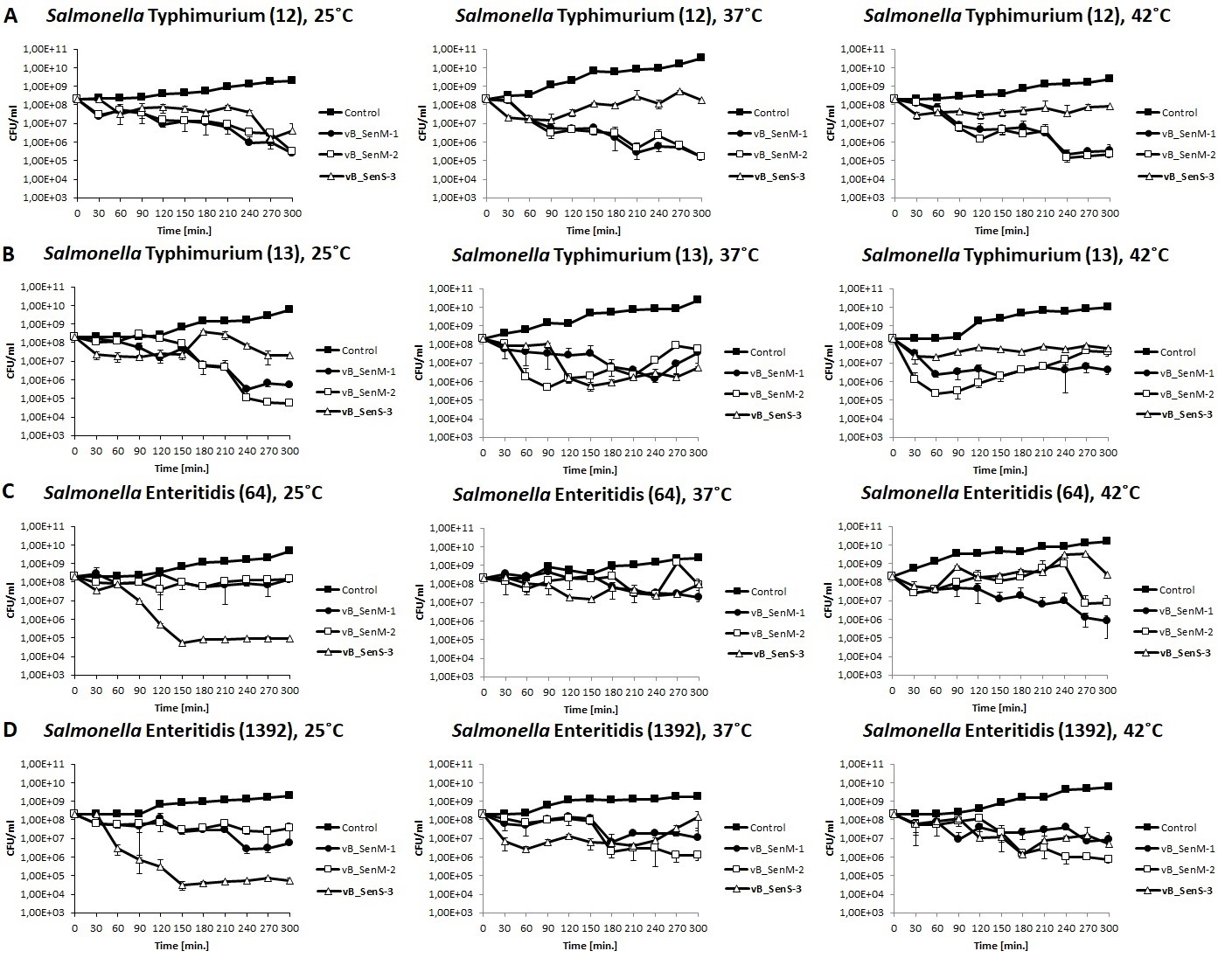

Supplement: Supplementary file 1 [file ijms-21-06152-s001.zip › SEN-KKK-Suppl-Figure S6-R1.jpg]

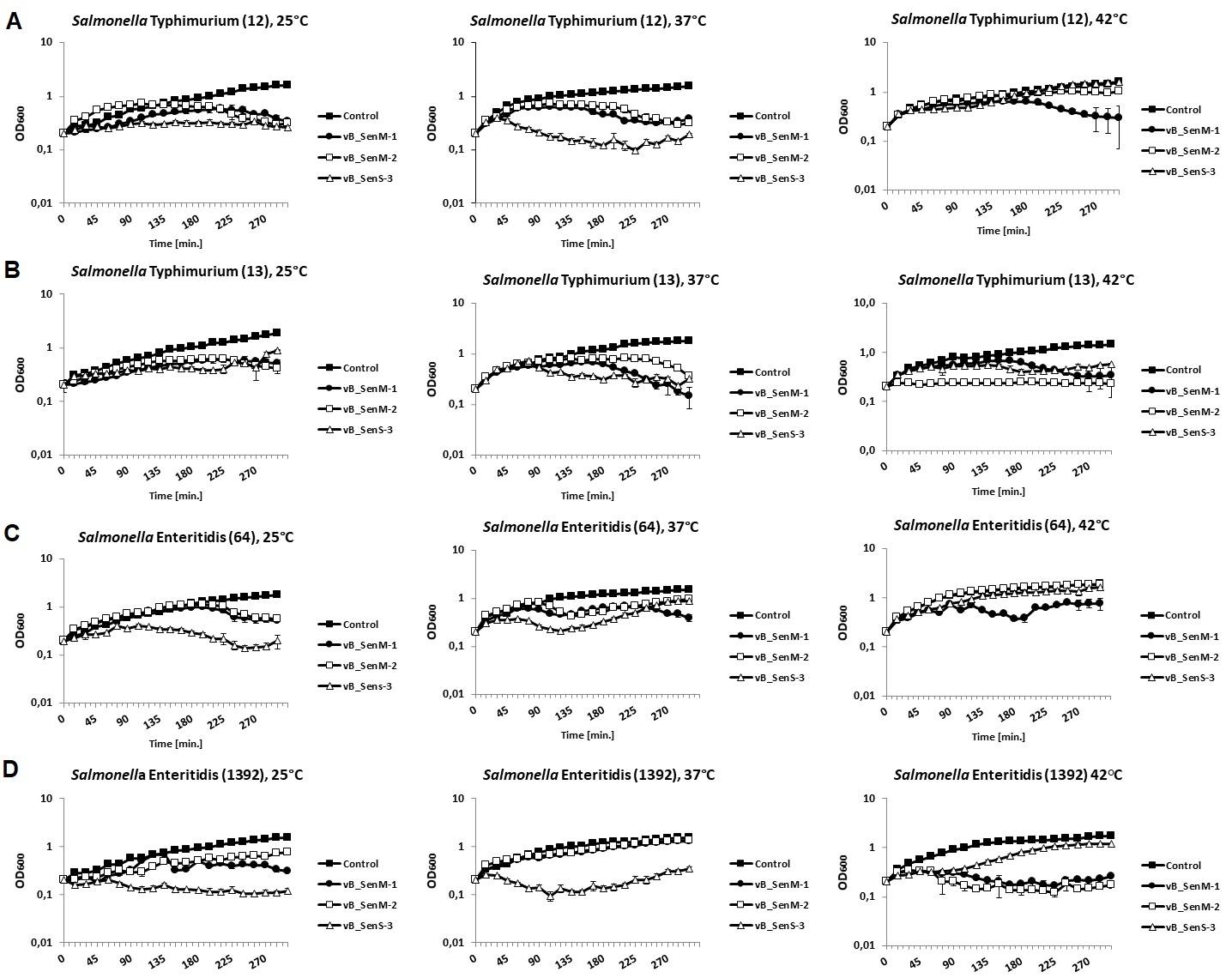

Supplement: Supplementary file 1 [file ijms-21-06152-s001.zip › SEN-KKK-Suppl-Figure S7-R1.jpg]

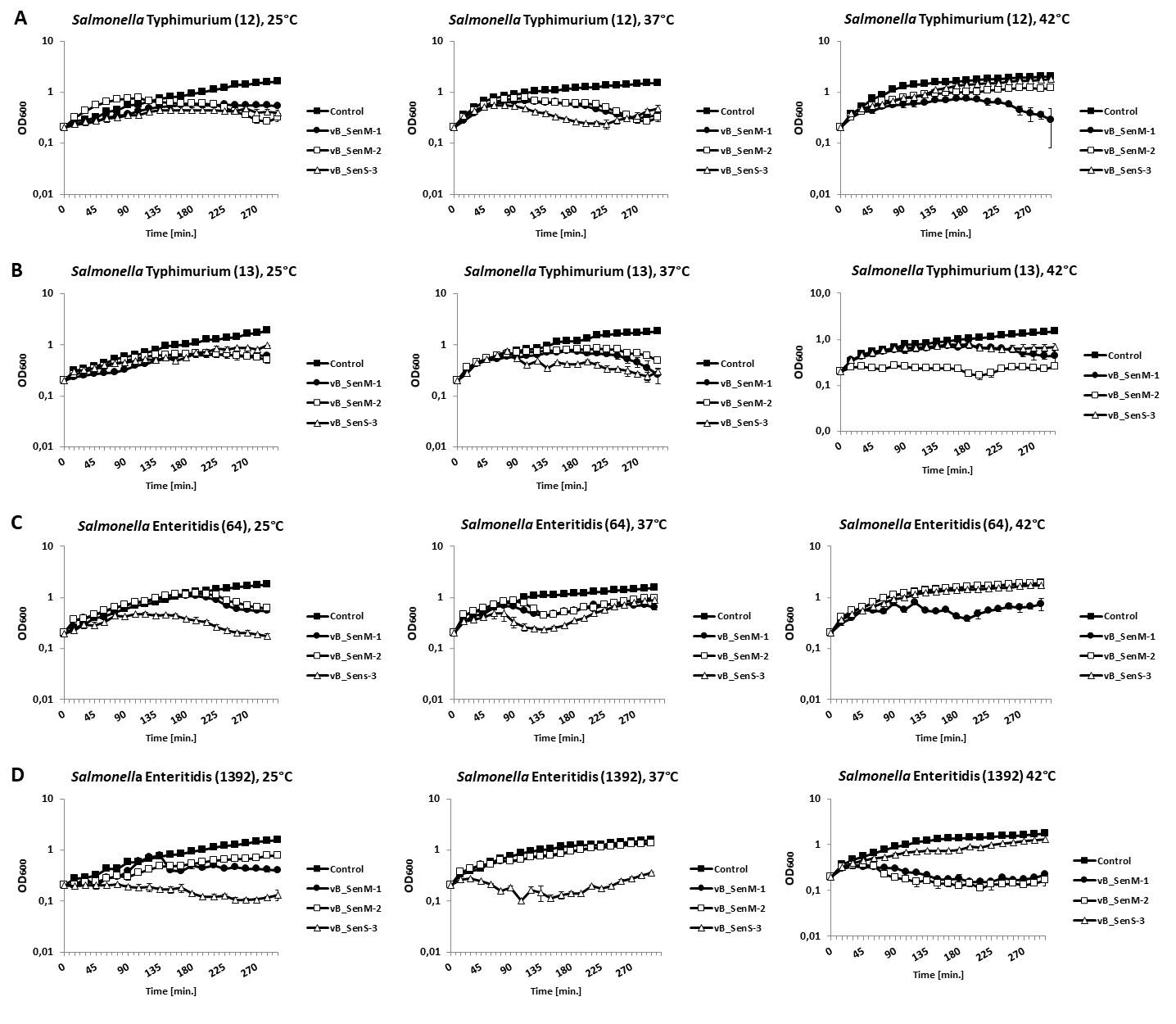

Supplement: Supplementary file 1 [file ijms-21-06152-s001.zip › SEN-KKK-Suppl-Figure S8-R1.jpg]

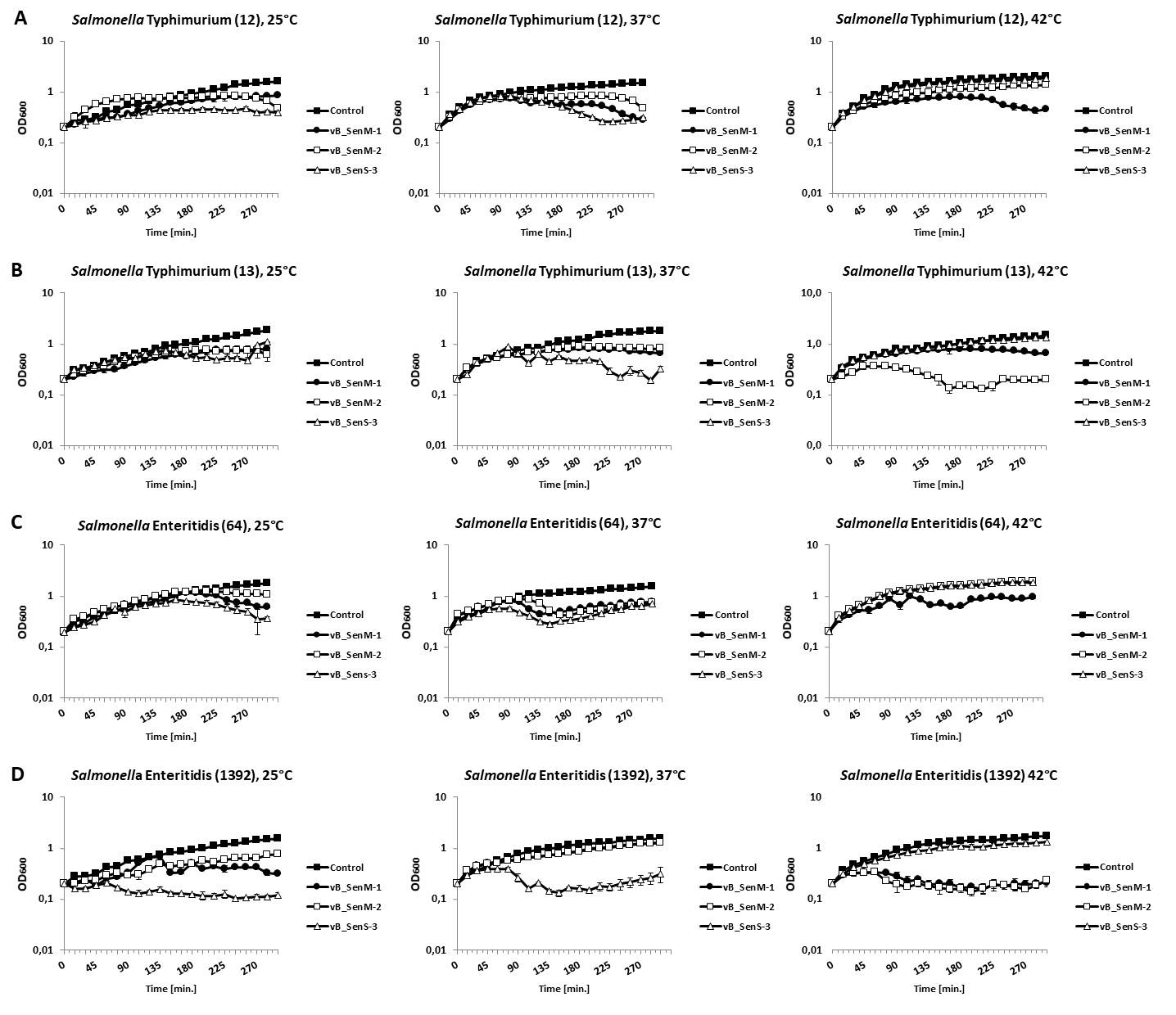

Supplement: Supplementary file 1 [file ijms-21-06152-s001.zip › SEN-KKK-Suppl-Figure S9-R1.jpg]
